# Mortality rates and cardiovascular disease burden in type 2 diabetes by occupation, results from all Swedish employees in 2002–2015

**DOI:** 10.1186/s12933-021-01320-8

**Published:** 2021-06-26

**Authors:** Sofia Carlsson, Tomas Andersson, Mats Talbäck, Maria Feychting

**Affiliations:** 1 Institute of Environmental Medicine, Karolinska Institutet, 171 77 Stockholm, Sweden; 2 Centre for Occupational and Environmental Medicine, Stockholm County Council, Stockholm, Sweden

**Keywords:** Type 2 diabetes, Occupation, Workplace intervention, Mortality, Cardiovascular disease, Incidence, Epidemiology

## Abstract

**Objective:**

To identify occupations where employees with type 2 diabetes have a high risk of cardiovascular disease (CVD) and mortality, and their prevalence of CVD risk factors. This study can contribute in the creation of targeted interventions at the workplace.

**Research design and methods:**

This nationwide registry-based study included all employees with type 2 diabetes born in Sweden in 1937–1979 (n = 180,620) and followed up in 2002–2015. We calculated age-standardized incidence (per 100,000 person-years) of all-cause and CVD mortality, ischemic heart disease (IHD) and stroke across the 30 most common occupations. Information on prognostic factors was retrieved from the National Diabetes Register.

**Results:**

In males with type 2 diabetes, mortality rates were highest in manufacturing workers (1782) and machine operators (1329), and lowest in specialist managers (633). The risk of death at age 61–70 years was 21.8% in manufacturing workers and 8.5% in managers. In females with type 2 diabetes, mortality rates were highest in manufacturing workers (1150) and cleaners (876), and lowest in writers and artists (458); the risk of death at age 61–70 years was 12.4% in manufacturing workers and 4.3% in writers and artists. The same occupations also had relatively high incidences of CVD mortality, IHD and stroke. Occupational groups with poor prognosis had high prevalence of CVD risk factors including poor glycemic control, smoking and obesity.

**Conclusions:**

Manufacturing workers, machine operators and cleaners with type 2 diabetes have two to three times higher mortality rates than managers, writers and artists with type 2 diabetes. Major health gains would be made if targeted workplace interventions could reduce CVD risk factors in these occupations.

**Supplementary Information:**

The online version contains supplementary material available at 10.1186/s12933-021-01320-8.

## Introduction

Type 2 diabetes is associated with serious complications, with cardiovascular disease (CVD) being the main cause of morbidity and mortality [1]. Because of its many comorbidities, diabetes is a major burden on the healthcare system [2]. In addition, type 2 diabetes has adverse effects on work ability, as it is associated with increased sickness absence and early retirement [3, 4]. Importantly, the risk of vascular complications can be reduced through a combination of lifestyle modifications and pharmacological interventions [5]. Major health and societal gains would be made if efficient secondary prevention could improve the prognosis for the large and growing number of people with type 2 diabetes [6, 7]. The workplace is a potentially important and underused arena for prevention; workplace interventions have shown promising results [8] and adults spend a large proportion of their time at work. The first step in such an approach is to identify occupations in which employees with type 2 diabetes have a high prevalence of CVD risk factors and are at high risk of adverse outcomes.

Several studies have shown that low socioeconomic status (SES) is associated with increased risk of CVD and mortality in individuals with type 2 diabetes [9, 10], but studies of specific occupations are lacking. Regarding the risk of developing type 2 diabetes, we recently reported a large variation across occupational groups; manufacturing workers, professional drivers and cleaners had 2–3 times higher rates of the disease than university teachers and physiotherapists [11]. The differences are far greater than those seen across socioeconomic groups [12]. We also found that occupations with high type 2 diabetes incidence were characterized by high prevalence of overweight, smoking and low physical fitness [11]. This could put employees with type 2 diabetes in these occupations at high risk of CVD. Furthermore, workplace factors such as irregular working hours, shift work, stress and physically strenuous work may hinder optimal self-management and contribute to poor glycemic control and excess risk of vascular complications in some occupations [13–16]. At present, it is not clear if and how the prognosis of people with type 2 diabetes differs across occupational groups.

To address this knowledge gap, we investigated the risk of all-cause mortality and CVD mortality and morbidity in males and females with type 2 diabetes across the 30 most common occupational groups among all people gainfully employed in Sweden. The aim was to identify occupations where employees with type 2 diabetes have a high risk of CVD and mortality and to describe their prevalence of key CVD-risk factors.

## Research design and methods

### Registry linkage

This nationwide study was based on the Prescribed Drug Register (PDR), the National Patient Register (NPR), the Cause of Death Register, the National Diabetes Register (NDR) and the Longitudinal integration database for health insurance and labour market studies (LISA by its Swedish acronym) (Additional file 1: Figure S1). The registers were linked using the personal identification numbers assigned to Swedish residents upon birth or immigration. The study was approved by the ethical review board in Stockholm (2017/706-31).

### Study population

We used LISA [17] to identify all individuals born in Sweden 1937–1979 and selected those who were gainfully employed at any time between 2001 and 2013 (n = 4,398,117; 2,245,231 males and 2,152,886 females); The birth years selected ensured that the participants were younger than 65 years in 2001 (the first year for which we had occupational information) and older than 35 years at the end of follow-up in 2015. This was our source population, in which we aimed to identify all people with type 2 diabetes.

### Diabetes

Prevalent cases of diabetes in 2001–2013 were identified through the NPR, the NDR and the PDR. The NPR contains information on all diagnoses from hospital admissions nationwide since 1987 and from outpatient specialist care since 2001, coded based on the Swedish version of the International Classification of Disease (ICD-10 since 1997) [18]. In the NPR, ICD codes E11 (type 2 diabetes) and E14 (unspecified diabetes) were used as indicators of type 2 diabetes. The PDR records all filled prescriptions since July 2005, based on the Anatomical Therapeutic Chemical (ATC) classification system [19]. ATC group A10 (insulin and oral antidiabetic drugs) was used to identify diabetes. If a case was identified solely through the PDR, age at first prescription ≥ 35 years was used as an indicator of type 2 diabetes. The NDR was created in 1996 with the aim of monitoring the health of people with diabetes [20]. The NDR records clinical characteristics for people over 18 years diagnosed with type 1, type 2 or gestational diabetes and covers 90% of all diabetes patients. In total, we identified 180,620 individuals with type 2 diabetes who were followed up for CVD and mortality. Data on the incidence and prevalence of type 2 diabetes by occupation in this population have been published previously [11].

### Occupation

Information on occupation was obtained by linkage to the LISA database [17]. LISA holds annual data from 1990 onwards, encompassing all Swedish citizens ≥ 16 years of age as of December 31 each year. Main occupation, i.e., the occupation with the highest taxable income, is recorded for each person in November each year. Occupations are classified based on the Swedish Standard Classification of Occupations 1996 (SSYK96) [21], which is a national version of the International Standard Classification of Occupations [22]. LISA also includes information on highest attained level of education.

### Risk factors

We used the NDR to collect information on CVD risk factors in individuals with type 2 diabetes for the period 2001–2013. This included information on duration of diabetes, glycated hemoglobin A1c (HbA_1c_), albuminuria, systolic blood pressure, low-density lipoprotein (LDL) cholesterol, estimated glomerular filtration rate (eGFR, using the Modification of Diet in Renal Disease equation), body mass index (BMI), smoking and physical activity. We used all information recorded while the person was gainfully employed. Information from NDR was available for 71% of males with type 2 diabetes and 67.2% of females with type 2 diabetes from the time when they were employed. No corresponding information was available for people without diabetes.

### Outcomes

People with type 2 diabetes were followed for mortality and cardiovascular morbidity between 2002 and 2015 in the NPR and the Cause of Death Register. We used the outcomes all-cause mortality, CVD mortality (ICD-10 codes I00–I99), ischemic heart disease (ICD-10: I20–I25) and stroke (ICD10: I61–I64) (including fatal). Additionally, we followed the entire source population, consisting of all those who were gainfully employed in 2001–2013, for the same outcomes.

### Statistical analyses

In the SSYK96 [21], there are 113 occupational groups at the three-digit level. We chose to focus on the 30 most common occupations in males and females, respectively, which were selected based on the total number of person-years of follow-up in each occupation. We calculated age-standardized mortality and CVD incidence from 2002 to 2015 and 95% confidence intervals (CIs) across occupational groups in people with type 2 diabetes. Employees were followed from the first recording indicating T2D in any of the registers. Incidence is expressed per 100,000 person-years in this paper. The age distribution of the total employed population with type 2 diabetes from 2002 to 2015 was used for weighting. Since there were few observations in some occupations at certain ages, we standardized by use of a multivariable logistic model for each occupation, with age and calendar year as explanatory variables in the model; age was included as a third-degree polynomial function. We applied the estimated model to the total employed population with type 2 diabetes to predict annual incidence of the outcome event. All analyses were run separately in males and females. Standardized incidence ratios (SIRs) were also calculated for each occupation compared with the total employed population with type 2 diabetes. We estimated the 10-year risk of all-cause mortality, CVD mortality, IHD and stroke at age 60 years by occupation in people with type 2 diabetes conditioned on being alive at that age. The risk were calculated as the complement to the estimated probability of being event-free through ages 61 to 70 years in 2015, using the age and occupation specific cumulative incidence from the same model for standardization. Based on the 10-year risk at age 60 years, we also estimated the attributable risk percentage (AR%) for each occupation using the formula (RR − 1)/RR, where RR is the relative risk in each occupation using the occupation with the lowest risk as reference. This number shows the proportion of cases that would be eliminated if the risk in a specific occupation was equal to that in the occupation with the lowest risk. The analyses were repeated in the source population consisting of the total employed population (Additional file 1: Tables S10–S13).

### CVD risk factors

CVD risk factors in people with type 2 diabetes across occupational groups were presented as mean values and standard deviations for continuous variables. We also calculated the proportion within each occupational group that was above a target level for each risk factor. In line with current treatment guidelines [23, 24], we used the cut-off > 7.0% (53 mmol/mol) for HbA_1c_, > 140 mmHg for systolic blood pressure, > 2.5 mmol/l for LDL cholesterol, and BMI ≥ 30 kg/m^2^ for obesity. In addition, albuminuria was defined as either microalbuminuria (2/3 positive results, i.e., a urinary albumin/creatinine ratio of 3–30 mg/mmol or urinary albumin clearance of 20–200 μg/min within one year) or macroalbuminuria (a urinary albumin/creatinine ratio > 30 mg/mmol or urinary albumin clearance > 200 μg/min), low physical activity as being active for at least 30 min less than once per week, and smoking as smoking at least one cigarette per day.

## Results

### Characteristics

**Fig. 1:**
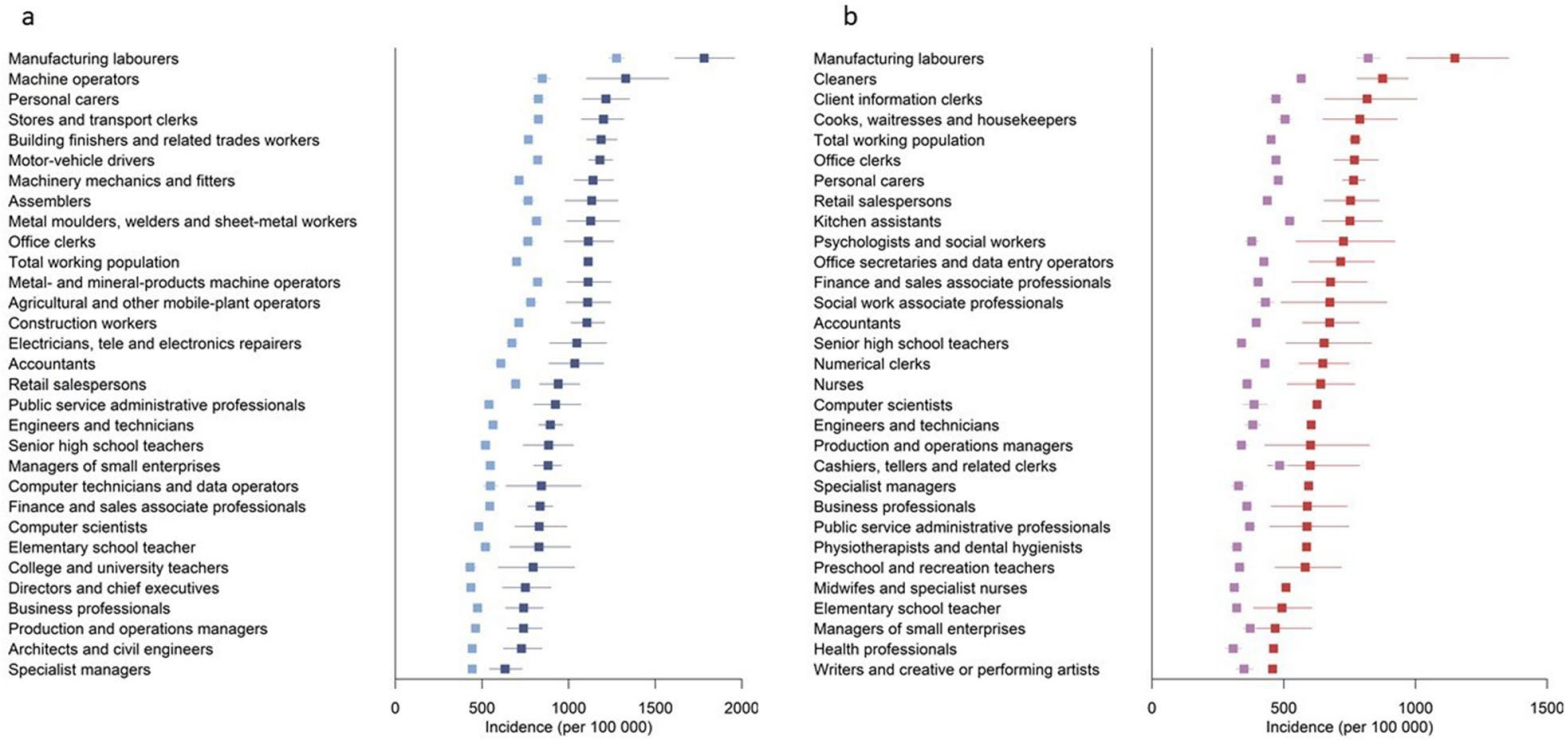
**a** Age-standardized all-cause mortality (per 100,000 person-years) from 2002 to 2015 across the 30 most common occupations in Swedish males. Blue squares, males with type 2 diabetes; light blue squares, all males. **b** Age-standardized all-cause mortality (per 100,000 person-years) from 2002 to 2015 across the 30 most common occupations in Swedish females. Red squares, females with type 2 diabetes; light purple squares, all females

**Fig. 2:**
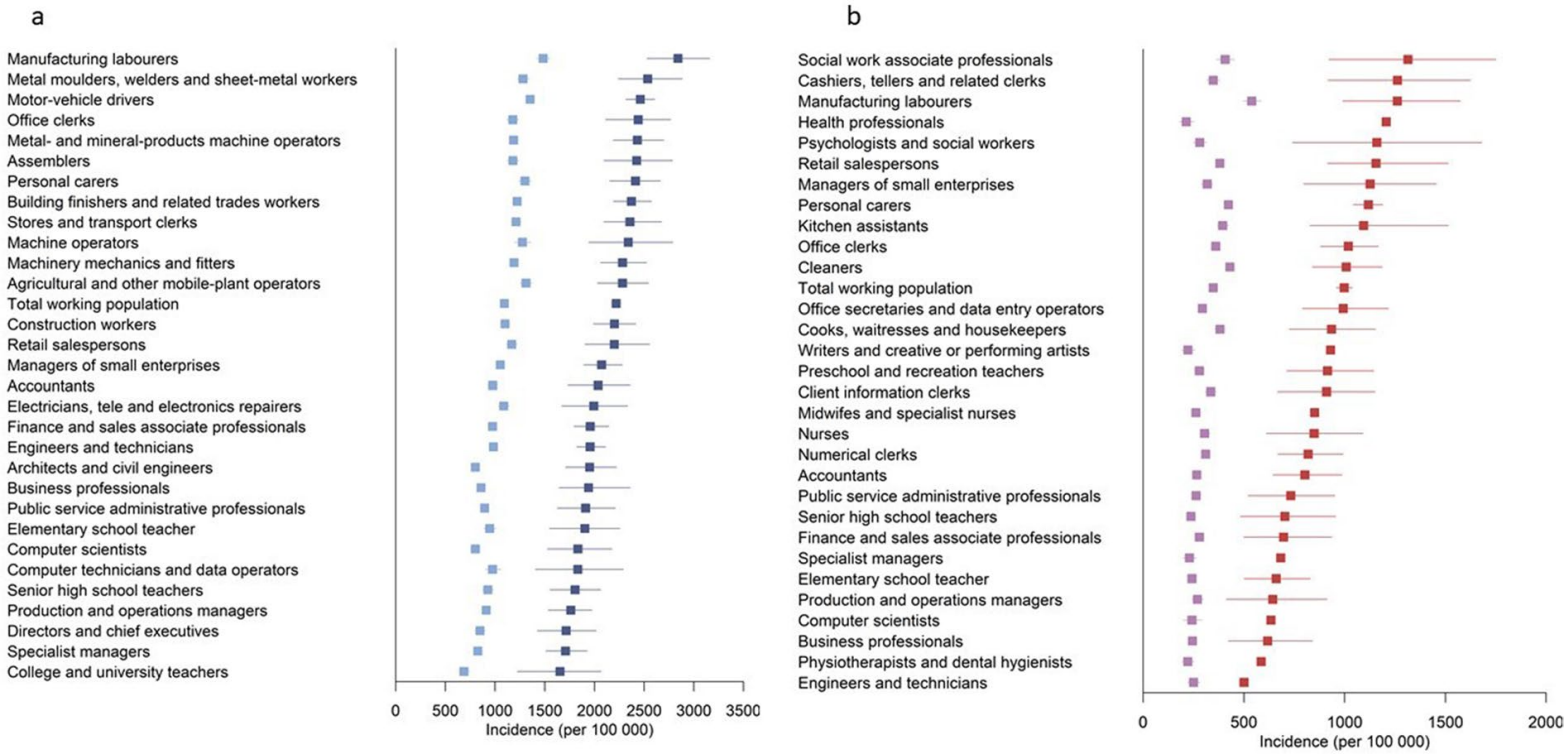
**a** Age-standardized incidence of ischemic heart disease (per 100,000 person-years) from 2002 to 2015 across the 30 most common occupations in Swedish males. Blue squares, males with type 2 diabetes; light blue squares, all males. **b** Age-standardized incidence of ischemic heart disease (per 100,000 person-years) from 2002 to 2015 across the 30 most common occupations in Swedish females. Red squares, females with type 2 diabetes; light purple squares, all females

There were 180,620 individuals with type 2 diabetes, among whom there were 14,881 deaths, including 4661 from CVD, during 1.5 million years of follow-up. In the general population, there were 167,575 deaths over 45.7 million years of follow-up. Incidence of all-cause mortality, CVD mortality, IHD and stroke was higher in individuals with type 2 diabetes than in the general population, in both males and females, and this difference was seen across all occupational groups (Figs. 1 and 2, Additional file 1: Figures S2–S5, Tables S2, S3). The risk of death at age 61–70 years was 13.8% in males and 10.0% in females with type 2 diabetes, compared with 8.7% and 5.6%, respectively, in the general population. Males had higher mortality, IHD and stroke incidence than females, and this difference was seen in those with type 2 diabetes as well as in the general population (Additional file 1: Table S1).

### Males with type 2 diabetes

**Fig. 3:**
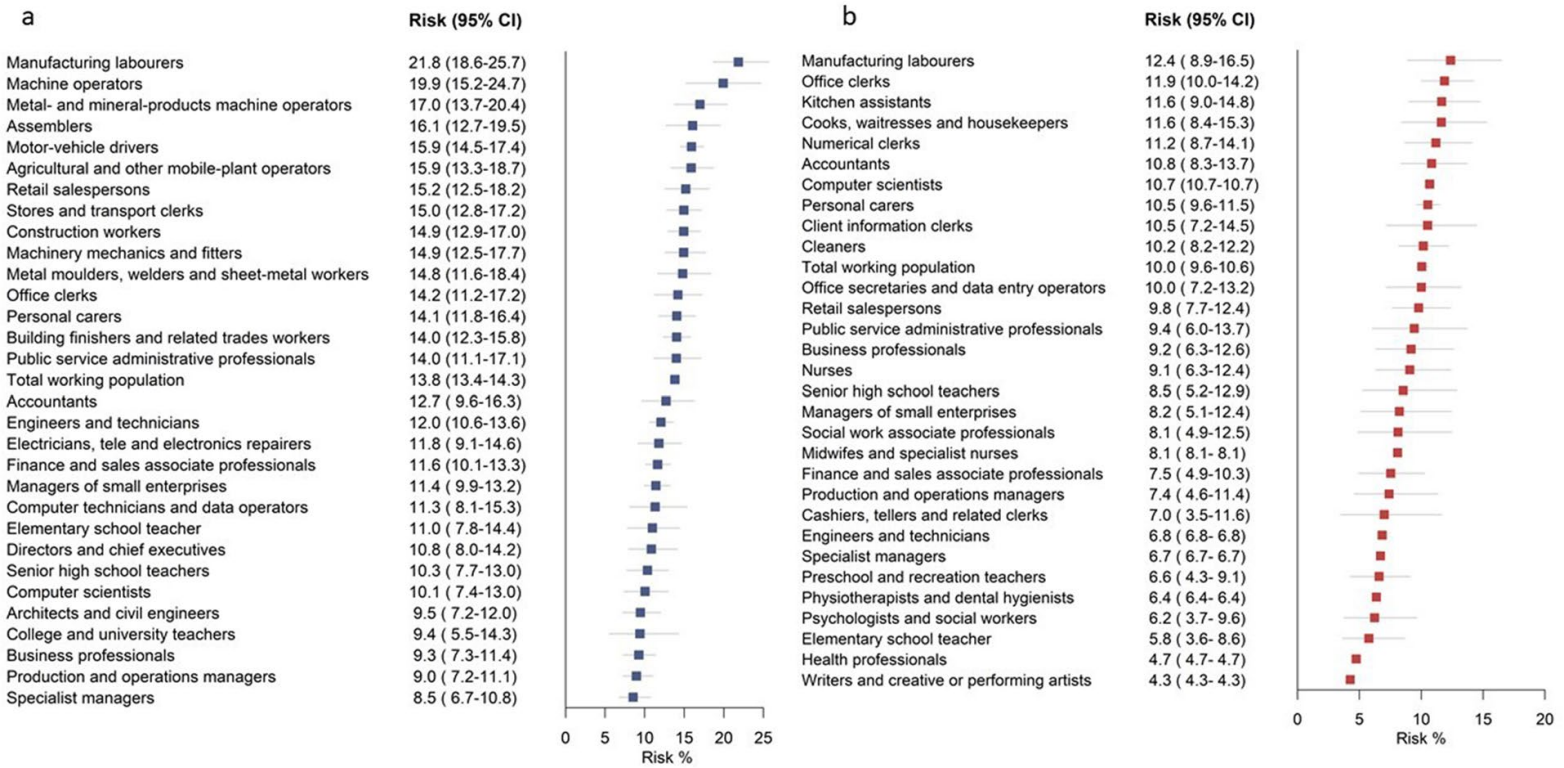
**a** The risk (per 100 persons) of dying at age 61–70 years in males with type 2 diabetes across the 30 most common occupations in Swedish males. **b** The risk (per 100 persons) of dying at age 61–70 years in females with type 2 diabetes across the 30 most common occupations in Swedish females

In males with type 2 diabetes, all-cause mortality rates (per 100,000 person-years) were highest in manufacturing workers (1782), machine operators (1329) and personal carers (1215), and lowest in specialist managers (633) (Fig. 1, Additional file 1: Table S4). The same occupational groups also had the highest and lowest CVD mortality rates, respectively (Additional file 1: Figure S2). SIR calculations indicated that manufacturing workers had 1.59 (95% CI 1.44–1.76) times higher mortality rates compared with the total employed population with type 2 diabetes and specialist managers had 43% (SIR 0.57, 95% CI 0.49–0.66) lower mortality rates. The 10-year risk of death at age 60 years was 21.8% in manufacturing workers and 19.9% in machine operators compared with 8.5% in specialist managers (Fig. 3﻿, Additional file 1: Table S4). Estimation of AR% indicated that 61% of all deaths at age 61–70 years among manufacturing workers with type 2 diabetes would be eliminated if they had the same mortality risk as specialist managers with type 2 diabetes (Additional file 1: Table S4).

The incidence of IHD was highest in manufacturing workers (2814) and metal molders, welders, and sheet-metal workers (2536), and lowest in college/university teachers (1653) (Fig. 2 and Additional file 1: Table S5). Stroke incidence was highest in motor vehicle drivers (771), followed by manufacturing workers (753), and lowest in computer technicians/data operators (447) (Additional file 1: Figure S3 and Table S5). The risk of IHD at age 61–70 years was 24.7% in manufacturing workers compared with 16.9% in college/university teachers. Motor vehicle drivers and manufacturing workers had a 9.6% risk of a stroke event at age 61–70 years; the risk among computer technicians/data operators was 4.5%.

### Females with type 2 diabetes

In females with type 2 diabetes, mortality rates (per 100,000 person-years) were highest in manufacturing workers (1150) and cleaners (876), and lowest in health professionals (461) and writers/artists (458) (Fig. 1, Additional file 1: Table S6). SIR calculations indicated that manufacturing workers with type 2 diabetes had 1.53 times higher and writers/artists had 41% lower mortality rates than the general population with type 2 diabetes. Similar findings were seen for CVD mortality (Additional file 1: Figure S4, Table S6). The risk of death at age 61–70 years in females with type 2 diabetes was highest in manufacturing workers (12.4%), office clerks (11.9%), and kitchen assistants (11.6%), and lowest in writers/artists (4.3%) (Fig. 3, Additional file 1: Table S6). Estimation of AR% indicated that 65.5% of all deaths at age 61–70 years in manufacturing workers with type 2 diabetes would be eliminated if they had the same mortality risk as writers/artists with type 2 diabetes (Additional file 1: Table S6).

Incidence of IHD was highest in social work professionals (1314; this includes youth recreation leaders and treatment assistants), manufacturing workers (1262), and cashiers and tellers (1262), and lowest in engineers and technicians (501) (Fig. 2, Additional file 1: Table S7). The risk of IHD at age 61–70 years was 11.5% in manufacturing workers, 11.1% in social work professionals, and 7.8% in engineers and technicians. The incidence of stroke was highest in manufacturing workers (534)—six times higher than in health professionals (87) (Additional file 1: Figure S5 and Table S7)—with the risk of being affected at age 61–70 years being 4.6% in manufacturing workers and 1.6% in health professionals.

In both males and females with type 2 diabetes, the incidence was highest in occupations characterized by low SES (Additional file 1: Tables S4–S7) according to the classification used by Statistics Sweden [25], including both skilled and unskilled manual laborers (occupations that require less than 12 years’ education).

### CVD risk factors in males and females with type 2 diabetes

**Fig. 4:**
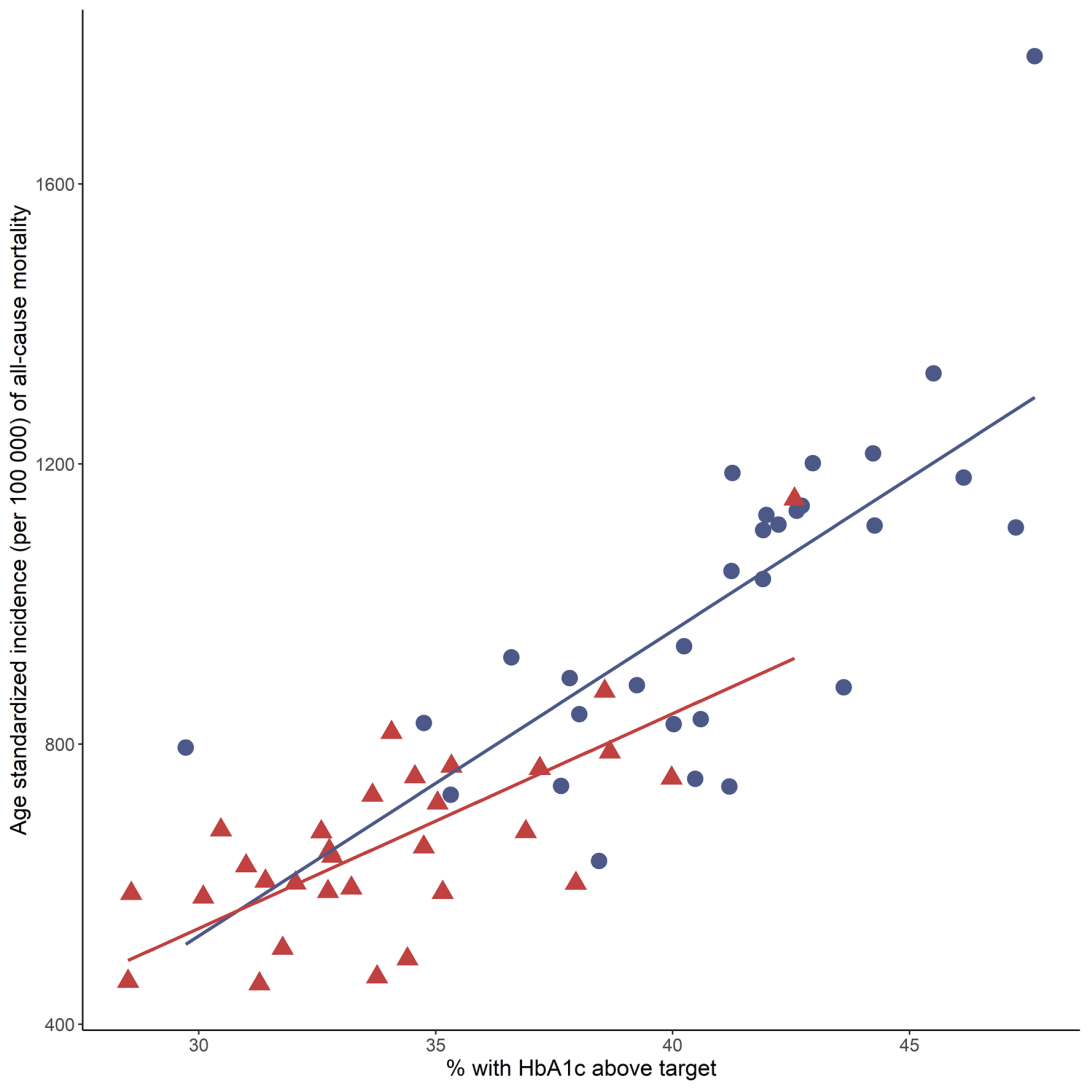
Proportion (%) with HbA_1c_ levels above target (> 7.0% (53 mmol/mol)) and age-standardized all-cause mortality (per 100,000 person-years) from 2002 to 2015 in people with type 2 diabetes, across the 30 most common occupations in Swedish males and females. Blue circles, males; red triangles, females

Mean duration of type 2 diabetes was 6.3 years in females and 6.4 years in males and did not seem to vary by occupation (Additional file 1: Tables S8, S9). Occupations with high mortality and CVD rates were characterized by higher prevalence of CVD risk factors than occupations with low rates (Additional file 1: Tables S8, S9). As an example, male manufacturing workers had a higher prevalence of smoking (25.1 vs. 10.0%, *p* < 0.0001), obesity (50.7 vs 42.5%, *p* < 0.0001), albuminuria (24.3 vs 19.6%, *p* 0.0030) and HbA_1c_ levels above 7% (53 mmol/mol) (47.6 vs. 38.4%, *p* < 0.0001) than male specialist managers. Similar results were seen when comparing female manufacturing workers to female writers/artists regarding smoking (25.6 vs. 12.7%, *p* < 0.0001), albuminuria (16.8 vs 8.9%, *p* 0.0109) and HbA_1c_ levels above 7.0% (53 mmol/l) (42.6 vs 31.3%, *p* 0.0030). Regarding LDL cholesterol, systolic blood pressure and low physical activity, the variation across high- to low-risk occupations was less consistent. As shown in Fig. 4, there was a clear positive association between all-cause mortality and the proportion with HbA_1c_ levels above target within the 30 most common occupations for males and females. Similar associations with HbA_1c_ were seen for incidence of CVD mortality, IHD and stroke (Additional file 1: Figures S6–S8).

### The total employed population

Among the total male employed population, the highest all-cause and CVD mortality, stroke and IHD incidence was seen in manufacturing workers, followed by machine operators (mortality) and motor vehicle drivers (IHD and stroke) (Figs. 1 and 2, Additional file 1: Figures S2, S3, Tables S10, S11). All-cause mortality, CVD mortality, IHD and stroke incidence in the total female employed population was highest in manufacturing workers followed by cleaners (Figs. 1, 2, Additional file 1: Figures S4, S5 and Tables S12, S13).

## Discussion

This nationwide descriptive study revealed large variation in all-cause mortality, CVD mortality, and the incidence of IHD and stroke among people with type 2 diabetes across the 30 most common occupational groups in Sweden. In females with type 2 diabetes, mortality rates ranged from 458–1150 per 100,000 person-years and were highest in manufacturing workers and cleaners and lowest in health professionals and writers/artists. In males with type 2 diabetes, mortality rates were highest in manufacturing workers and machine operators and lowest in architects, civil engineers, and specialist managers, ranging from 633 to 1782 per 100,000 person-years. The same occupations had high CVD mortality, IHD and stroke incidence. Occupations associated with poor type 2 diabetes prognosis were characterized by higher prevalence of CVD risk factors, including poor glycemic control, smoking, obesity, and low physical activity.

As far as we know, this is the first study to give a comprehensive overview of the prognosis of people with type 2 diabetes across occupational groups. However, a Chinese study found lower death rates in nurses with diabetes than the general population of patients [26], which is in line with our findings. Previous studies indicate that low SES is associated with higher rates of mortality and CVD in individuals with type 2 diabetes [9, 10]. In support of this, we found that occupations where employees with type 2 diabetes had a poor prognosis were characterized by low SES. However, our analysis showed large variation between occupational groups within the low socioeconomic stratum, e.g., CVD mortality rates were 33% higher in male manufacturing workers with type 2 diabetes than in male machine operators and assemblers with type 2 diabetes, and 77% higher in female manufacturing workers with type 2 diabetes than in female cleaners with type 2 diabetes.

Previous studies based on general working populations found the highest death rates among factory workers, cleaners, and construction workers [27–29]. We could confirm these findings in our population and extend them by showing that having type 2 diabetes increased the risk even further in these already vulnerable groups, e.g., manufacturing workers with type 2 diabetes had about twice the IHD rates and 40% higher mortality rates than the general population of manufacturing workers. Previous findings based on the Swedish National Diabetes Register indicate that smoking, physical inactivity and HbA_1c_ levels above target are the strongest predictors of mortality in type 2 diabetes [30]. In line with this, we observed that these risk factors were more prevalent in the occupations where people with type 2 diabetes had the poorest prognosis.

We have previously demonstrated that manufacturing workers, cleaners and machine operators have higher incidence of type 2 diabetes than other occupational groups and higher prevalence of overweight, smoking, and low physical fitness at young age (18–40 years) [11]. In the present study, we show that if such workers develop type 2 diabetes, they have a poorer prognosis than their counterparts in other occupational groups, including 2–3 times higher mortality rates and high incidence rates of IHD and stroke. Out of all occupational groups, manufacturing workers fared worst: they had the highest type 2 diabetes risk and the poorest prognosis. In absolute terms, the risk of death at age 61–69 years in manufacturing workers with type 2 diabetes was 21.8% (males) and 12.4% (females), compared with 8.5% in specialist managers (males) and 4.3% in writers/artists (females). Manufacturing workers were also most often smokers and most often had albuminuria and HbA_1c_ levels and systolic blood pressure above target. Targeted interventions seem warranted, and the preventive potential appears substantial; estimations of AR% indicate that > 60% of all deaths at age 61–70 years among manufacturing workers with type 2 diabetes would be eliminated if they had the same death risk as workers with diabetes in low-risk occupations. This is particularly important as type 2 diabetes is common, affecting 14.9% of male and 10.7% of female manufacturing workers 55 years or older in Sweden [11]. It should be noted that the job title serves as a risk indicator in this context, and we are not proposing that occupation per se is a causal factor in relation to diabetes complications.

Efficient first and secondary prevention that reduces the risk of type 2 diabetes and its complications would benefit public health and could lead to major healthcare and productivity gains. A US study from 2016 estimated that diabetes accounted for the largest healthcare costs out of all medical conditions [31]. Moreover, a recent Swedish study found that costs related to being absent from work exceeded those for hospital-based care [32]. Vascular complications are preventable [5] and there are studies showing that workplace interventions, including supervised group sessions promoting healthy eating, physical activity, and self-management in employees with type 2 diabetes, may improve diabetes outcomes, including HbA_1c_ levels [8]. In this context, it should be noted that adaptations of diabetes prevention programs to workplace settings have also shown promising results [33]. There is a close relationship between T2D and several CVDs including heart failure [34] and arterial fibrillation [35] and once both conditions are present, mortality is increased substantially. Ideally, all employees in occupations where the risks of type 2 diabetes and CVD are high should be targeted with interventions to increase physical activity, reduce weight gain, and promote smoking cessation. This could reduce the risk of both conditions. Further research in this area is warranted, both for confirmation of our findings and for addressing aspects that we did not cover, including the burden of sickness absence, disability pension and early retirement attributable to diabetes in different occupational groups, the risk of microvascular complications by occupation, and the extent to which working conditions affect the prognosis of individuals with type 2 diabetes.

The strengths of this study included the nationwide design that comprised the entire Swedish employed population and information collected from national registers, with virtually no loss to follow-up. Information on occupation came from a national database that is updated annually and has a 95% completeness [17]. Diabetes cases were identified through a combination of patient, prescription, and diabetes registers. Since the PDR can be expected to cover all pharmacologically treated patients and the NDR covers > 90% of all patients [20], we have probably identified most cases. However, undiagnosed diabetes will be missed, and the proportion of such cases may differ between occupations. Type 2 diabetes diagnosis was based on ICD code or age ≥ 35 years at the time of the first prescription of diabetes medication. This means that some individuals with adult-onset autoimmune diabetes will be included. As this is relatively rare, it is unlikely to affect the results significantly. In addition, the NDR provided us with detailed information on CVD risk factors, including HbA_1c_ levels, for most individuals with type 2 diabetes. Limitations include the lack of information on CVD risk factors in the general population, which would have been valuable for comparison of risk factor patterns. We also lacked information on working conditions, such as stress, working hours, shift work and physical demands, which may influence the risk of mortality and CVD and contribute to differences in risk factor patterns. There may also be differences in antidiabetic and cardioprotective treatment across occupational groups which could affect prognosis. The NPR and cause of death register were used for outcome assessment. The validity of these registers is high for most diagnoses, including myocardial infarction and stroke [18, 36]. The extent to which our findings are generalizable to other countries with larger socio-economic differences and other working conditions than in Sweden remains to be seen. It should be noted that our findings regarding the general employed population were in line with those of previous reports from the UK [27], Belgium [28] and Australia [29], indicating high mortality in manufacturing workers. Moreover, Sweden’s overall diabetes prevalence is in line with that in Europe as a whole [7].

Our findings indicate that job title can be used to find the groups that would benefit most from targeted workplace interventions. Hopefully, this knowledge can inspire employers whose employees have high risk of type 2 diabetes and poor prognosis if affected by type 2 diabetes to implement healthy lifestyle interventions to improve the health of their workforce.

## Supplementary Information

**Additional file 1. **Additional tables and figures.

## Data Availability

The data that support the findings of this study are available from Statistics Sweden and the Swedish National Board of Health and Welfare, but restrictions apply to the availability of these data. They were used under license for the current study and are not publicly available.
