# Supplementary material for: Mortality rates and cardiovascular disease burden in type 2 diabetes by occupation, results from all Swedish employees in 2002–2015
Source: Cardiovasc Diabetol. 2021 Jun 26;20:129. doi: 10.1186/s12933-021-01320-8 (PMC8235252; doi:10.1186/s12933-021-01320-8)
Supplement: Supplementary file 1 — Additional file 1. Additional tables and figures. [file 12933_2021_1320_MOESM1_ESM.docx]

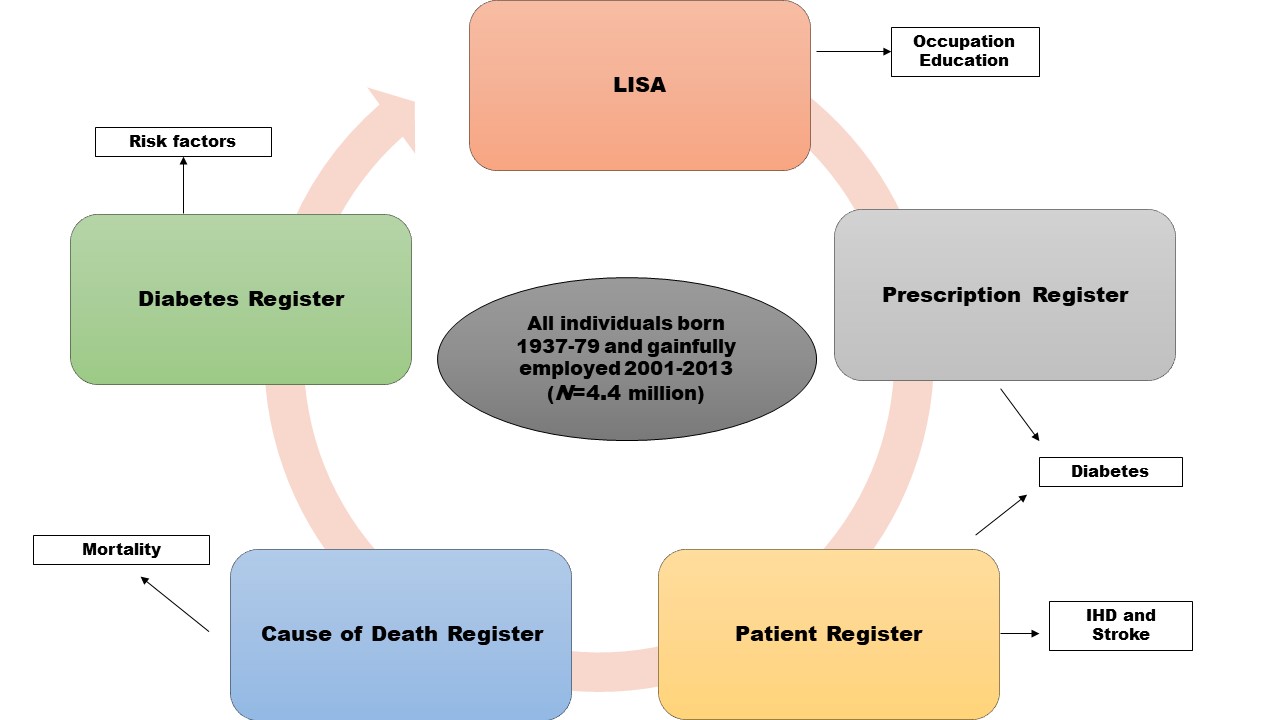


figure S1. Linkage between national registers.

table S1. Characteristics of all Swedish citizens born 1937-79 who were gainfully employed 2001-2013.

|  | ****General population**** | | | | | ****Individuals with type 2 diabetes**** | | | |  |
| --- | --- | --- | --- | --- | --- | --- | --- | --- | --- | --- |
|  | **Men** | **Women** | | **Men** | | | **Women** | |  |  |
| **No.** | 2 245 231 | 2 152 886 | | 115 931 | | | 64 689 | |  |  |
| ****Age at baseline No (%)**** |  |  | |  | | |  | |  |  |
| **35-44** | 1 285 493 (57.3) | 1 227 083 (57.0) | | 13 211 (11.4) | | | 9 793 (15.1) | |  |  |
| **45-54** | 556 070 (24.8) | 542 040 (25.2) | | 39 820 (34.3) | | | 21 168 (32.7) | |  |  |
| **≥55** | 403 668 (18.0) | 383 763 (17.8) | | 62 900 (54.3) | | | 33 728 (52.1) | |  |  |
|  |  |  | |  | | |  | |  |  |
| ****Education No (%)**** |  |  | |  | | |  | |  |  |
| **Primary school** | 416 247 (18.5) | 290 941 (13.5) | | 33 068 (28.5) | | | 14 071 (21.8) | |  |  |
| **Secondary school** | 1 077 379 (48.0) | 1 002 820 (46.6) | | 55 966 (48.3) | | | 33 289 (51.5) | |  |  |
| **University** | 728 943 (32.5) | 848 456 (39.4) | | 26 396 (22.8) | | | 17 101 (26.4) | |  |  |
| **Missing** | 22 662 (1.0) | 10 669 (0.5) | | 501 (0.4) | | | 228 (0.4) | |  |  |
|  |  |  | |  | | |  | |  |  |
| ****Country of origin No (%)**** |  |  | |  | | |  | |  |  |
| **Born in Sweden** | 1 920 239 (85.5) | 1 832 320 (85.1) | | 94 875 (81.8) | | | 52 175 (80.7) | |  |  |
| **Born in Europe outside Sweden** | 194 117 (8.6) | 199 675 (9.3) | | 11 840 (10.2) | | | 7 293 (11.3) | |  |  |
| **Born outside Europe** | 130 875 (5.8) | 120 891 (5.6) | | 9 216 (7.9) | | | 5 221 (8.1) | |  |  |
|  |  |  | |  | | |  | |  |  |
| ****All-cause mortality**** |  |  | |  | | |  | |  |  |
| ****P**erson years** | 23 157 642 | 22 552 280 | | 963 250 | | | 540 199 | |  |  |
| **No. deaths** | 101 458 | 66 117 | | 10 716 | | | 4 165 | |  |  |
| **Age standardized mortality per 100 000 (95% CI)** | 700 (695-706) | 452 (448-456) | | 1 113 (1 092-1 133) | | | 771 (748-793) | |  |  |
|  |  |  | |  | | |  | |  |  |
| ****CVD mortality**** |  |  | |  | | |  | |  |  |
| **No. deaths** | 29 440 | 10 641 | | 3 695 | | | 966 | |  |  |
| **Age standardized mortality per 100 000 (95% CI)** | 210 (207-213) | 77 (75-78) | | 384 (371-397) | | | 179 (167-190) | |  |  |
|  | | |  | |  | | |  | | |
| ****Ischemic heart disease**** | | |  | |  | | |  | | |
| **No. events** | 160 891 | 51 534 | | 21 372 | | | 5 391 | |  |  |
| **Age standardized incidence per 100 000 (95% CI)** | 1 094 (1 085-1 103) | 348 (343-353) | | 2 219 (2 174-2 262) | | | 998 (959-1 038) | |  |  |
|  |  |  | |  | | |  | |  |  |
| ****Stroke**** |  |  | |  | | |  | |  |  |
| **No. events** | 52 705 | 28 068 | | 5 968 | | | 1 949 | |  |  |
| **Age standardized incidence per 100 000 (95% CI)** | 373 (369-377) | 195 (192-198) | | 620 (602-637) | | | 361 (343-380) | |  |  |
|  |  |  | |  | | |  | |  |  |

**
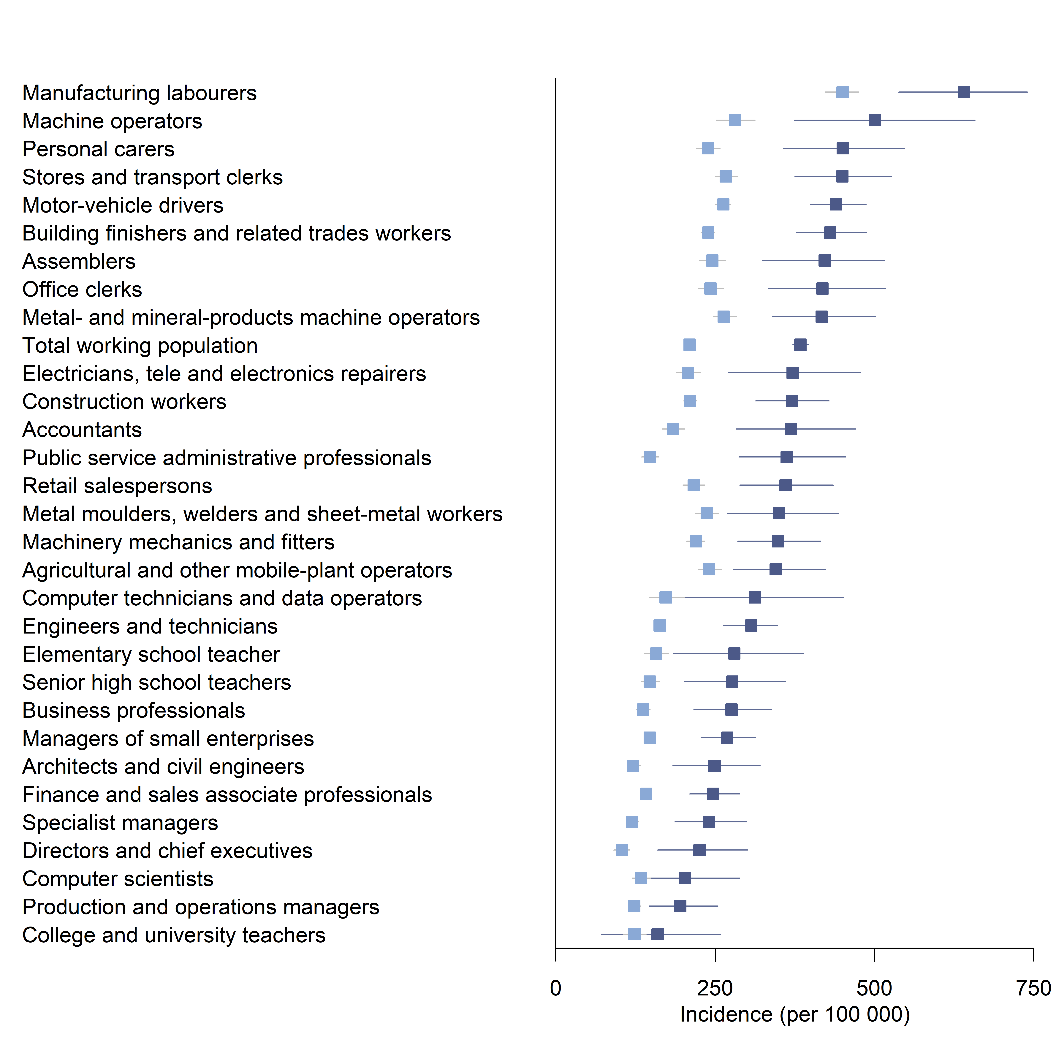
**

Figure S2. Age-standardized CVD mortality (per 100 000 person-years) from 2002 to 2015 across the 30 most common occupations in Swedish men. Blue squares, men with TYPE 2 DIABETES; light blue squares, all men.


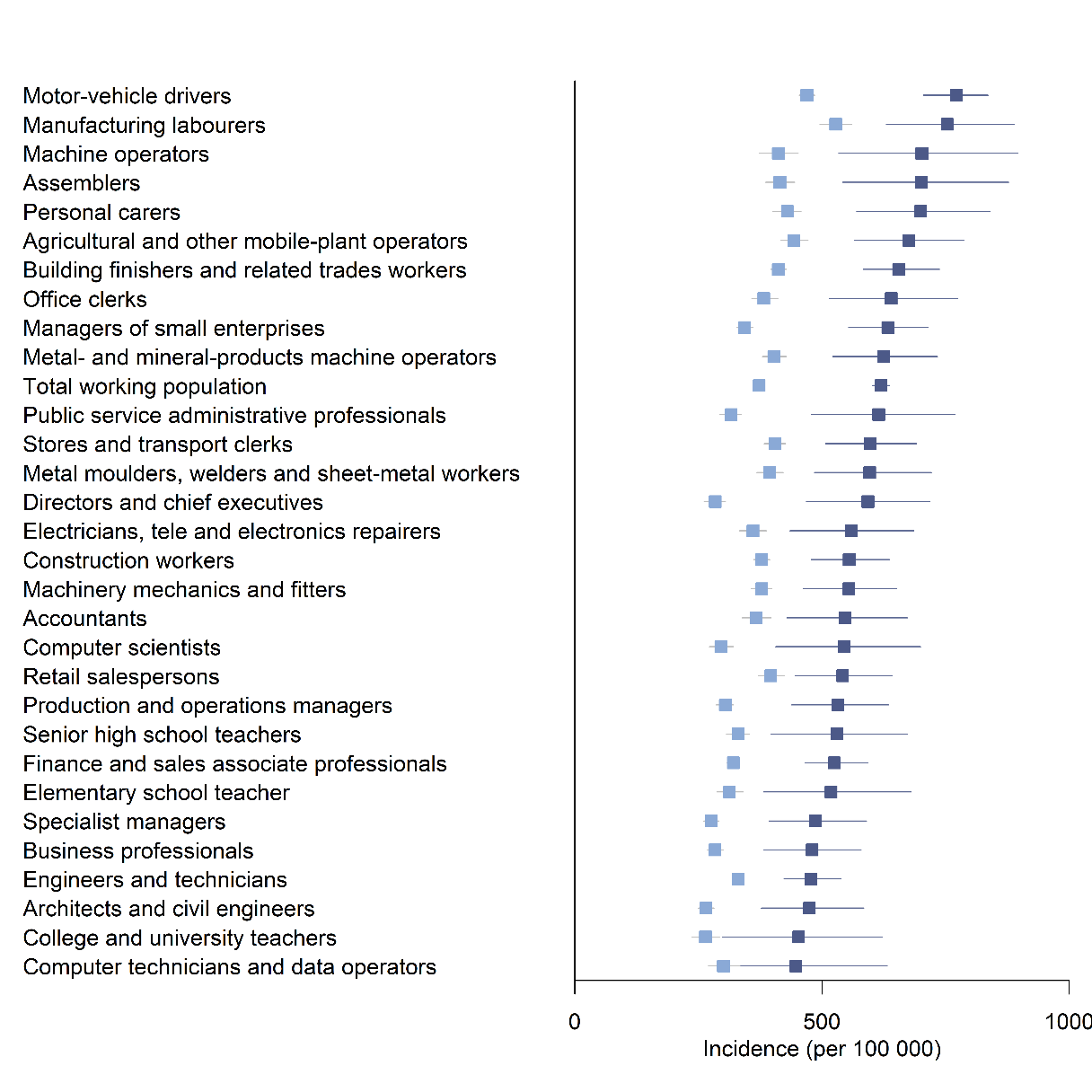


Figure S3. Age-standardized incidence (per 100 000 person-years) of Stroke From 2002 to 2015 across the 30 most common occupations in Swedish men. Blue squares, men with TYPE 2 DIABETES; light blue squares, all men.


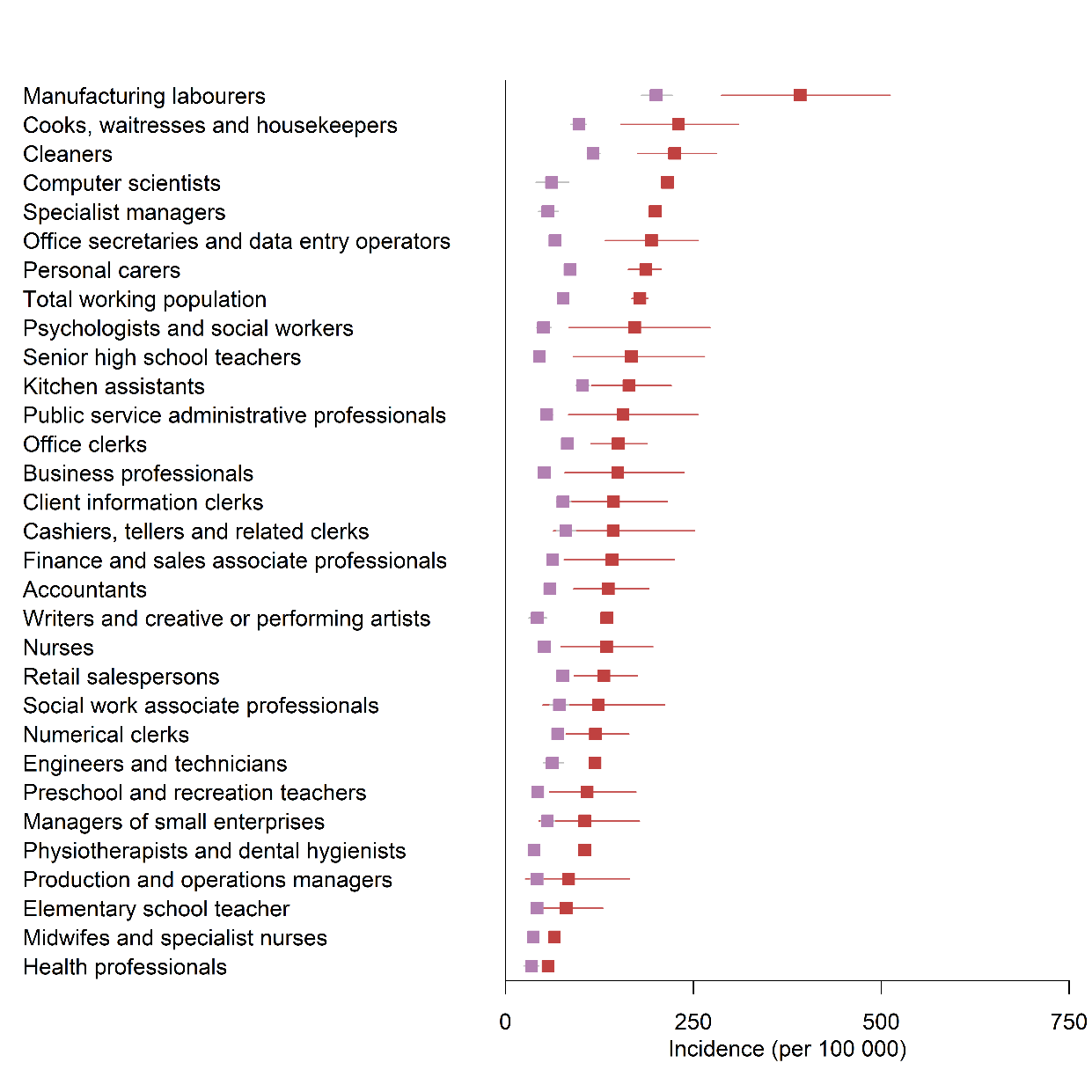


Figure S4. Age-standardized CVD mortality (per 100 000 person-years) from 2002 to 2015 across the 30 most common occupations in Swedish women. Red squares, women with TYPE 2 DIABETES; LIGHT purple squares, all women.


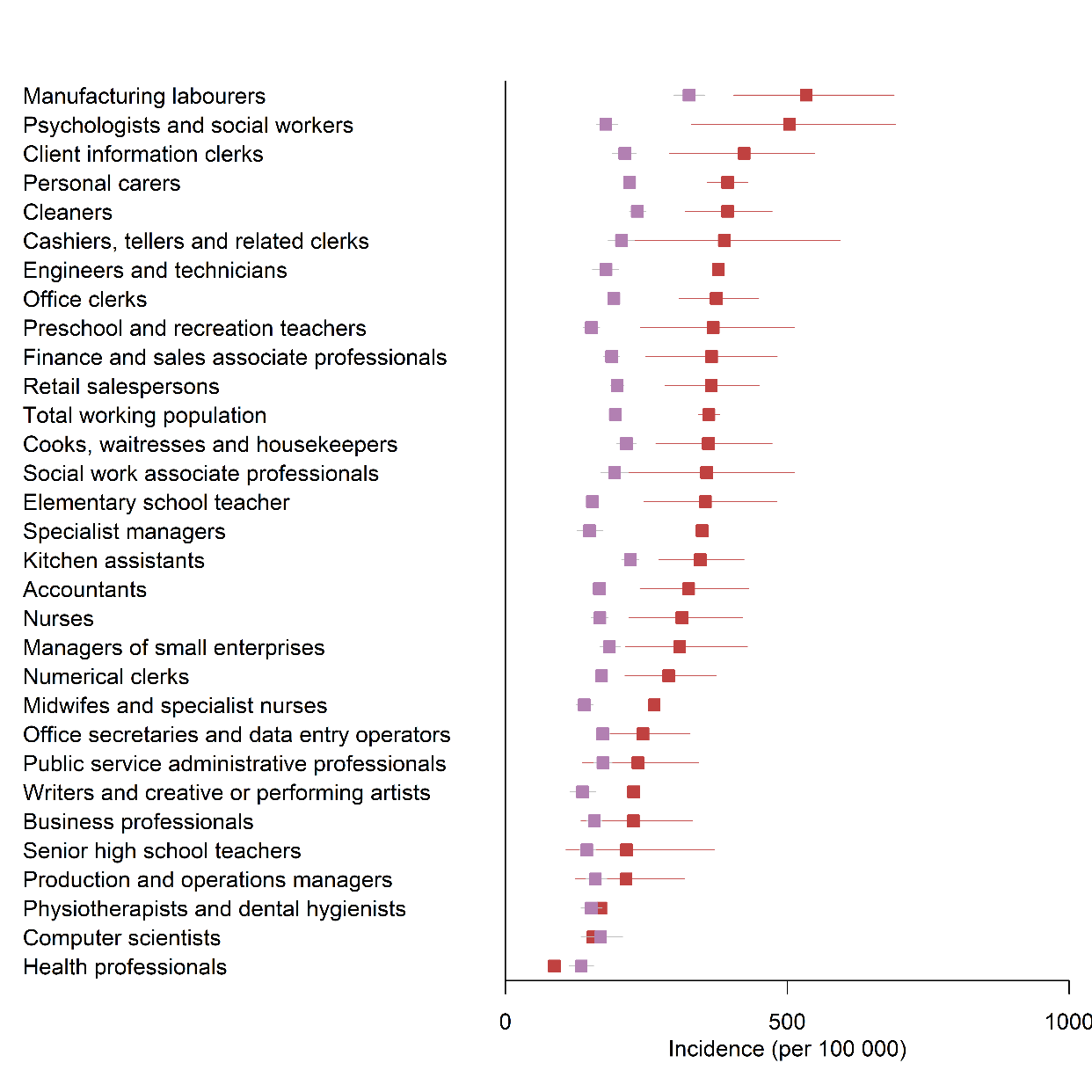


Figure S5. Age-standardized incidence (per 100 000 person-years) of stroke from 2002 to 2015 across the 30 most common occupations in Swedish women. Red squares, women with TYPE 2 DIABETES; Light purple squares, all women.

table S2. Difference in age-standardized incidence per 100 000 person-years (95% CI) in men with type 2 diabetes compared to all men by occupation.

|  | All-cause mortality | CVD-mortality | IHD | Stroke |
| --- | --- | --- | --- | --- |
| Manufacturing labourers | 5.06 (3.59-6.71) | 1.90 (0.97-2.94) | 13.58 (10.66-16.41) | 2.25 (1.08-3.55) |
| Machine operators | 4.82 (2.63-7.15) | 2.19 (0.94-3.65) | 10.66 (6.94-14.76) | 2.91 (1.25-4.67) |
| Personal carers | 3.90 (2.62-5.20) | 2.11 (1.26-3.00) | 11.13 (8.68-13.50) | 2.70 (1.45-4.07) |
| Stores and transport clerks | 3.76 (2.63-4.92) | 1.83 (1.12-2.57) | 11.46 (8.99-14.38) | 1.93 (1.09-2.86) |
| Building finishers and related trades workers | 4.20 (3.39-5.06) | 1.91 (1.43-2.45) | 11.51 (9.78-13.38) | 2.44 (1.75-3.22) |
| Motor-vehicle drivers | 3.60 (3.00-4.29) | 1.77 (1.39-2.20) | 11.09 (9.73-12.45) | 3.02 (2.41-3.63) |
| Machinery mechanics and fitters | 4.27 (3.24-5.37) | 1.29 (0.70-1.94) | 10.92 (8.81-13.33) | 1.76 (0.91-2.68) |
| Assemblers | 3.67 (2.17-5.17) | 1.76 (0.89-2.65) | 12.45 (9.27-15.99) | 2.86 (1.36-4.50) |
| Metal moulders. welders and sheet-metal workers | 3.13 (1.82-4.70) | 1.13 (0.36-2.00) | 12.55 (9.69-15.86) | 2.03 (0.97-3.27) |
| Office clerks | 3.49 (2.17-4.89) | 1.76 (0.94-2.68) | 12.62 (9.49-15.69) | 2.58 (1.34-3.86) |
| Total working population | 4.13 (3.93-4.32) | 1.74 (1.62-1.86) | 11.26 (10.83-11.68) | 2.47 (2.30-2.63) |
| Metal- and mineral-products machine operators | 2.92 (1.71-4.17) | 1.54 (0.79-2.33) | 12.45 (10.14-14.91) | 2.22 (1.19-3.24) |
| Agricultural and other mobile-plant operators | 3.28 (2.14-4.55) | 1.04 (0.41-1.78) | 9.71 (7.33-12.13) | 2.33 (1.28-3.39) |
| Construction workers | 3.94 (3.02-4.91) | 1.60 (1.07-2.17) | 11.02 (9.00-13.05) | 1.77 (1.02-2.54) |
| Electricians. tele and electronics repairers | 3.75 (2.27-5.36) | 1.64 (0.69-2.65) | 9.07 (6.03-12.29) | 1.99 (0.86-3.19) |
| Accountants | 4.27 (2.82-5.84) | 1.85 (1.03-2.79) | 10.61 (7.59-13.59) | 1.80 (0.67-3.01) |
| Retail salespersons | 2.46 (1.41-3.60) | 1.44 (0.75-2.18) | 10.32 (7.55-13.56) | 1.46 (0.52-2.39) |
| Public service administrative professionals | 3.84 (2.61-5.24) | 2.14 (1.43-3.00) | 10.17 (7.45-12.91) | 2.99 (1.65-4.46) |
| Engineers and technicians | 3.31 (2.66-3.99) | 1.44 (1.03-1.83) | 9.73 (8.39-11.19) | 1.48 (0.95-2.04) |
| Senior high school teachers | 3.64 (2.24-5.03) | 1.30 (0.58-2.09) | 8.77 (6.28-11.25) | 2.00 (0.71-3.36) |
| Managers of small enterprises | 3.33 (2.54-4.09) | 1.21 (0.81-1.63) | 10.19 (8.48-12.12) | 2.90 (2.15-3.64) |
| Computer technicians and data operators | 2.95 (1.00-5.16) | 1.40 (0.33-2.80) | 8.59 (4.57-12.97) | 1.47 (0.18-3.23) |
| Finance and sales associate professionals | 2.91 (2.22-3.57) | 1.06 (0.68-1.44) | 9.84 (8.25-11.54) | 2.04 (1.45-2.68) |
| Computer scientists | 3.49 (2.10-5.05) | 0.69 (0.02-1.55) | 10.32 (7.43-13.59) | 2.50 (1.17-3.97) |
| Elementary school teacher | 3.09 (1.45-4.91) | 1.23 (0.27-2.25) | 9.58 (6.11-12.85) | 2.05 (0.70-3.66) |
| College and university teachers | 3.64 (1.64-5.96) | 0.37 (-0.50-1.32) | 9.67 (5.53-13.59) | 1.88 (0.38-3.58) |
| Directors and chief executives | 3.15 (1.87-4.54) | 1.22 (0.58-1.97) | 8.67 (5.90-11.49) | 3.09 (1.87-4.31) |
| Business professionals | 2.66 (1.70-3.73) | 1.39 (0.82-2.00) | 10.81 (7.96-14.81) | 1.97 (1.04-2.92) |
| Production and operations managers | 2.77 (1.84-3.74) | 0.72 (0.25-1.30) | 8.50 (6.40-10.58) | 2.28 (1.40-3.25) |
| Architects and civil engineers | 2.84 (1.80-3.96) | 1.28 (0.65-1.98) | 11.50 (9.15-14.02) | 2.10 (1.15-3.16) |
| Specialist managers | 1.90 (1.01-2.84) | 1.21 (0.69-1.78) | 8.82 (6.85-10.87) | 2.10 (1.21-3.08) |

| Women | All-cause mortality | CVD-mortality | IHD | Stroke |
| --- | --- | --- | --- | --- |
| Manufacturing labourers | 3.29 (1.46-5.20) | 1.92 (0.85-3.08) | 7.23 (4.76-10.14) | 2.08 (0.81-3.58) |
| Cleaners | 3.09 (2.20-4.07) | 1.08 (0.59-1.61) | 5.77 (4.18-7.50) | 1.60 (0.88-2.35) |
| Client information clerks | 3.46 (1.90-5.26) | 0.67 (0.00-1.38) | 5.75 (3.34-8.15) | 2.12 (0.84-3.36) |
| Cooks. waitresses and housekeepers | 2.84 (1.50-4.24) | 1.33 (0.57-2.11) | 5.54 (3.61-7.65) | 1.46 (0.58-2.58) |
| Total working population | 3.19 (2.97-3.42) | 1.02 (0.91-1.13) | 6.50 (6.12-6.90) | 1.66 (1.49-1.85) |
| Office clerks | 2.98 (2.22-3.88) | 0.68 (0.31-1.05) | 6.58 (5.25-8.03) | 1.82 (1.18-2.55) |
| Personal carers | 2.86 (2.46-3.28) | 1.01 (0.79-1.21) | 6.95 (6.25-7.65) | 1.74 (1.38-2.08) |
| Retail salespersons | 3.16 (2.18-4.22) | 0.55 (0.16-1.01) | 7.75 (5.41-11.12) | 1.67 (0.90-2.52) |
| Kitchen assistants | 2.29 (1.22-3.48) | 0.62 (0.13-1.18) | 7.00 (4.48-10.95) | 1.24 (0.50-1.98) |
| Psychologists and social workers | 3.48 (1.72-5.40) | 1.21 (0.36-2.15) | 8.79 (4.74-13.84) | 3.26 (1.59-5.07) |
| Office secretaries and data entry operators | 2.91 (1.72-4.17) | 1.29 (0.69-1.91) | 6.99 (5.00-9.14) | 0.72 (-0.03-1.52) |
| Finance and sales associate professionals | 2.75 (1.32-4.13) | 0.79 (0.17-1.60) | 4.18 (2.28-6.56) | 1.78 (0.67-2.95) |
| Social work associate professionals | 2.44 (0.64-4.49) | 0.52 (-0.19-1.37) | 9.07 (5.22-13.27) | 1.63 (0.28-3.13) |
| Accountants | 2.79 (1.75-3.89) | 0.78 (0.32-1.30) | 5.37 (3.79-7.21) | 1.58 (0.74-2.63) |
| Senior high school teachers | 3.14 (1.67-4.99) | 1.22 (0.47-2.18) | 4.66 (2.51-7.15) | 0.70 (-0.35-2.18) |
| Numerical clerks | 2.19 (1.29-3.16) | 0.50 (0.12-0.94) | 5.09 (3.56-6.76) | 1.19 (0.44-2.01) |
| Nurses | 2.79 (1.50-4.09) | 0.83 (0.22-1.45) | 5.43 (3.09-7.87) | 1.46 (0.55-2.52) |
| Computer scientists | 2.39 (-0.88-7.08) | 1.54 (-0.25-7.21) | 3.92 (0.20-9.00) | -0.14 (-1.54-2.89) |
| Engineers and technicians | 2.22 (0.15-4.76) | 0.57 (-0.21-1.77) | 2.51 (0.45-4.96) | 2.00 (0.42-3.72) |
| Production and operations managers | 2.62 (0.83-4.90) | 0.42 (-0.13-1.21) | 3.74 (1.53-6.44) | 0.54 (-0.36-1.56) |
| Cashiers. tellers and related clerks | 1.17 (-0.44-3.00) | 0.63 (-0.13-1.66) | 9.15 (5.81-12.61) | 1.83 (0.13-3.89) |
| Specialist managers | 2.66 (0.38-5.53) | 1.43 (0.15-3.24) | 4.53 (1.32-8.92) | 2.00 (0.34-4.65) |
| Business professionals | 2.30 (0.98-3.79) | 0.98 (0.28-1.84) | 3.73 (1.82-6.03) | 0.70 (-0.19-1.73) |
| Public service administrative professionals | 2.17 (0.78-3.72) | 1.02 (0.33-2.00) | 4.70 (2.65-6.84) | 0.62 (-0.31-1.69) |
| Physiotherapists and dental hygienists | 2.64 (0.81-5.10) | 0.67 (-0.10-1.65) | 3.65 (1.06-6.57) | 0.17 (-0.97-1.76) |
| Preschool and recreation teachers | 2.49 (1.31-3.79) | 0.66 (0.17-1.30) | 6.36 (4.31-8.62) | 2.16 (0.88-3.56) |
| Midwifes and specialist nurses | 1.96 (0.60-3.46) | 0.28 (-0.15-0.96) | 5.89 (3.68-8.46) | 1.24 (0.28-2.39) |
| Elementary school teacher | 1.72 (0.65-2.81) | 0.39 (-0.02-0.87) | 4.18 (2.61-5.88) | 2.01 (0.95-3.26) |
| Managers of small enterprises | 0.95 (-0.23-2.30) | 0.50 (-0.10-1.22) | 8.09 (4.92-11.31) | 1.25 (0.25-2.44) |
| Health professionals | 1.53 (-0.37-3.96) | 0.22 (-0.30-1.12) | 9.92 (3.74-17.88) | -0.48 (-1.24-0.65) |
| Writers and creative or performing artists | 1.09 (-1.04-3.73) | 0.93 (-0.06-2.57) | 7.08 (2.20-12.52) | 0.91 (-0.57-2.67) |

table S3. Difference in age-standardized incidence per 100 000 person-years (95% CI) in men with type 2 diabetes compared to all men by occupation.

**
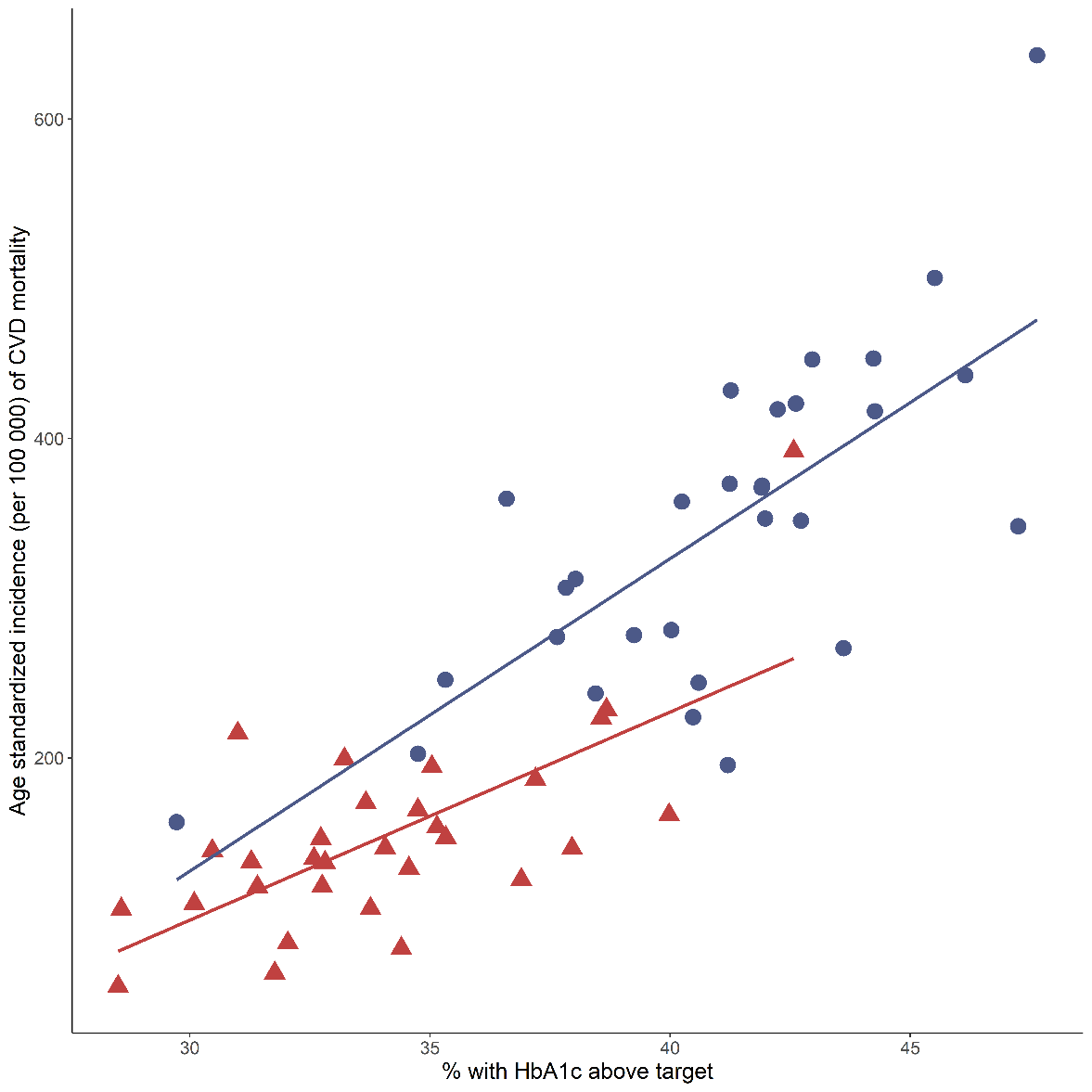
**

figure S6. Proportion (%) with HbA_1c_ above target ((>7.0% (53 mmol/mol)) and age-standardized incidence (per 100 000 person-years) of CVD Mortality from 2002 to 2015 in people with TYPE 2 DIABETES across the 30 most common occupations in Swedish men and women. Blue circles, men; red triangles, women.

**
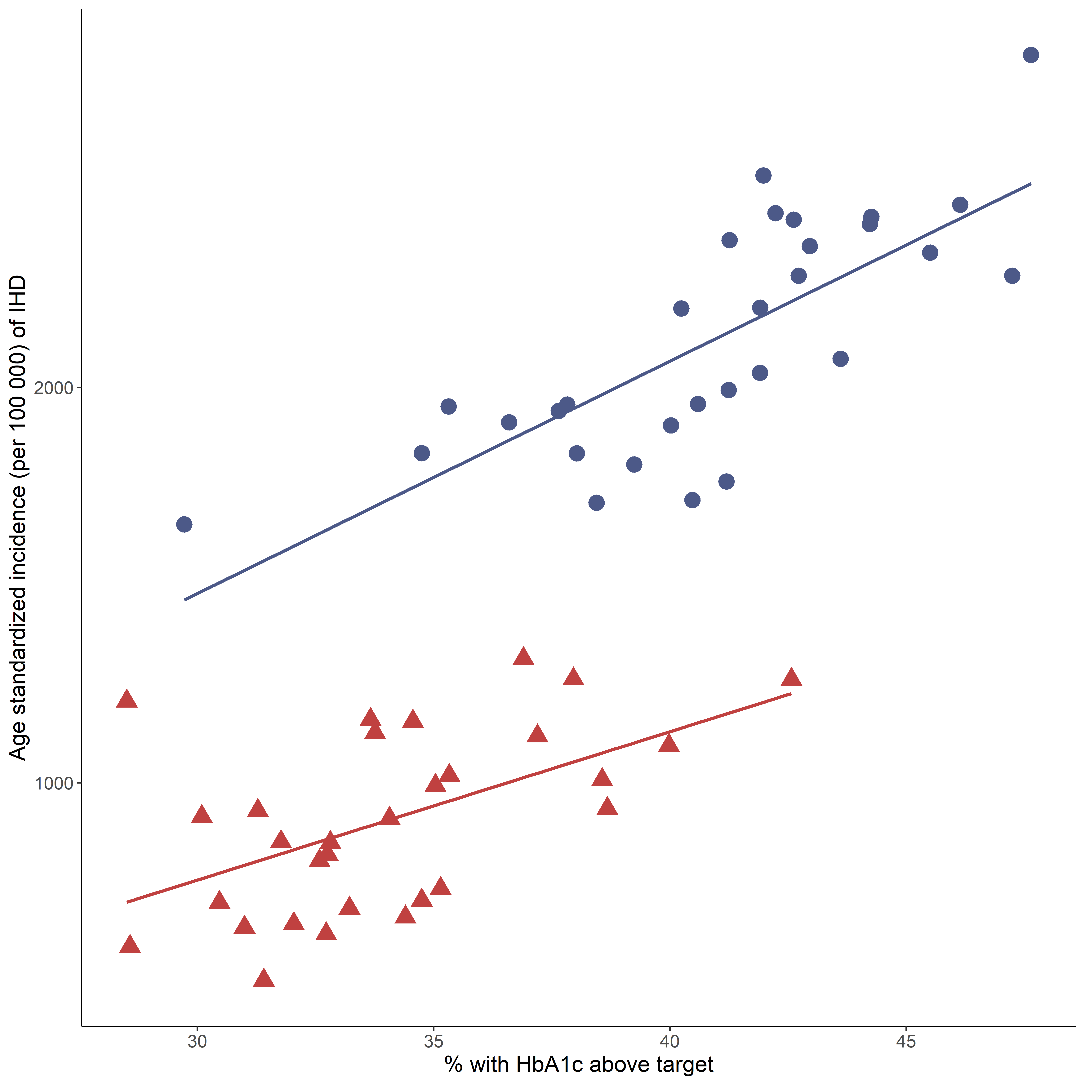
**

figure S7. Proportion (%) with HbA_1c_ above target (>7.0% (53 mmol/mol)) and age-standardized incidence (per 100 000 person-years) of IHD from 2002 to 2015 in people with TYPE 2 DIABETES across the 30 most common occupations in Swedish men and women. Blue circles, men; red triangles, women.


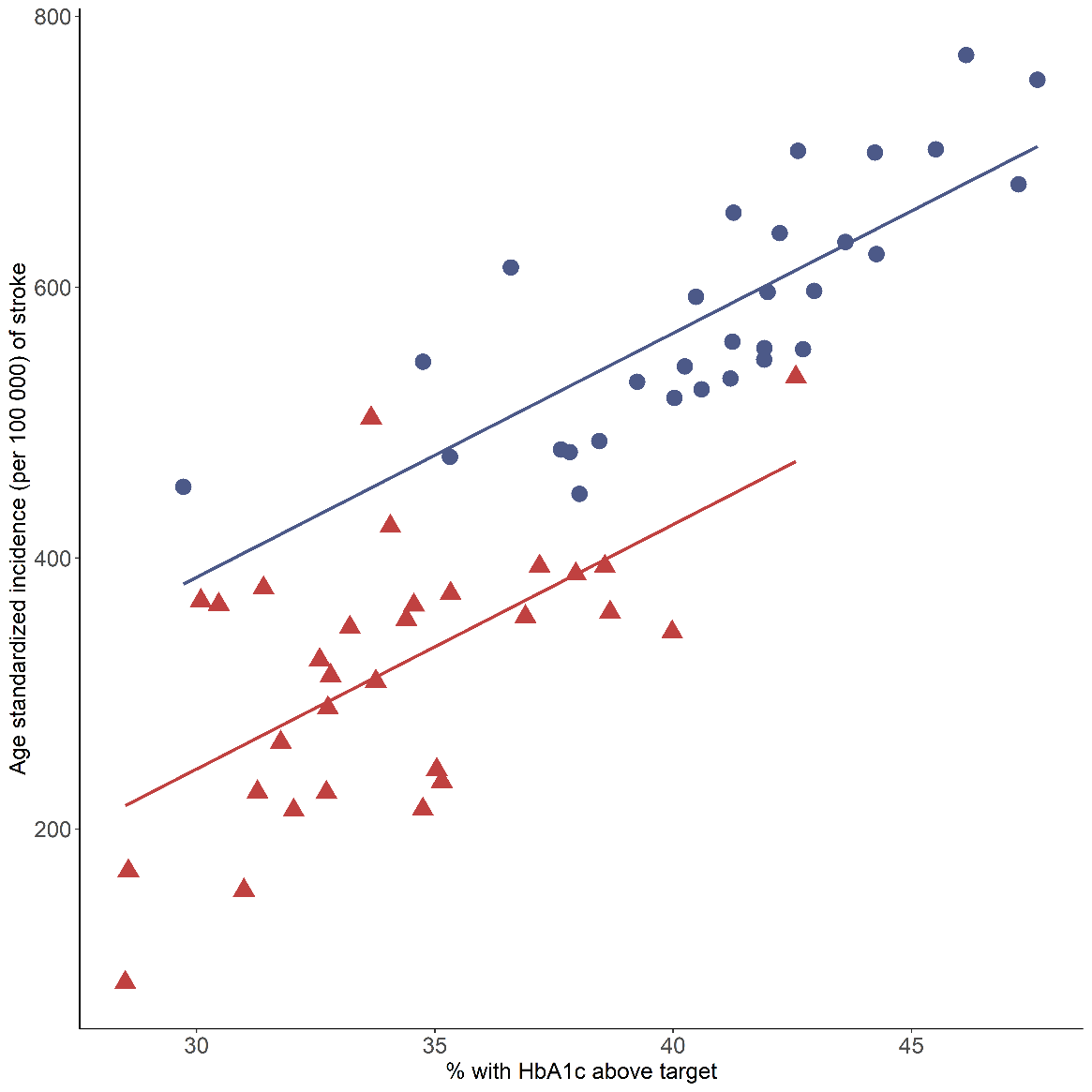
 Figure S8. Proportion (%) with HbA_1c_ above target (>7.0% (53 mmol/mol)) and age-standardized incidence (per 100 000 person-years) of stroke from 2002 to 2015 in people with TYPE 2 DIABETES across the 30 most common occupations in Swedish men and women. Blue circles, men; red triangles, women.

**Table S4. all cause and CVD mortality 2002-2015 in men with type 2 diabetes across the 30 most common occupations in Swedish men.**

|  | All-cause mortality | | | | | | | | CVD mortality | | | |  |
| --- | --- | --- | --- | --- | --- | --- | --- | --- | --- | --- | --- | --- | --- |
|  | **No. Cases** | **Person-years** | **Age stand per 100 000 (95% CI)** | **SIR**  **(95% CI)** | **Absolute**  **10-year risk at age 60** | **AR%^c^** | **No. Cases** | **Age stand per 100 000**  **(95% CI)** | | **SIR**  **(95% CI)** | **Absolute**  **10-year risk at age 60** | **AR%** |  |
|  |  |  |  |  |  |  |  |  |  |  |  |  |  |
| Manufacturing labourers^1^ | 402 | 23 615 | 1 782 (1 612-1 957) | 1.59 (1.44-1.76) | 21.8 (18.6-25.7) | 60.9 % | 144 | 640 (537-740) | | 1.65 (1.40-1.95) | 8.7 (6.4-11.3) | 76% |  |
| Machine operators^1^ | 139 | 12 124 | 1 329 (1 103-1 578) | 1.19 (1.00-1.41) | 19.9 (15.2-24.7) | 57.1% | 51 | 501 (374-658) | | 1.30 (0.97-1.71) | 9.3 (5.9-13.9) | 78% |  |
| Personal carers^1b^ | 327 | 35 768 | 1 215 (1 080-1 350) | 1.03 (0.92-1.14) | 14.1 (11.8-16.4) | 39.3% | 108 | 450 (357-547) | | 1.02 (0.83-1.23) | 5.2 (3.7-6.9) | 60% |  |
| Stores and transport clerks | 378 | 33 455 | 1 201 (1 074-1 318) | 1.08 (0.97-1.19) | 15.0 (12.8-17.2) | 42.9% | 142 | 450 (375-527) | | 1.18 (1.00-1.39) | 6.1 (4.6-7.8) | 66% |  |
| Building finishers and related trades workers^1^ | 689 | 59 659 | 1 187 (1 104-1 280) | 1.05 (0.98-1.14) | 14.0 (12.3-15.8) | 39.2% | 248 | 430 (378-487) | | 1.11 (0.97-1.25) | 4.6 (3.7-5.6) | 55% |  |
| Motor-vehicle drivers^1^ | 1 036 | 95 437 | 1 181 (1 115-1 254) | 1.05 (0.99-1.11) | 15.9 (14.5-17.4) | 46.4% | 383 | 440 (399-487) | | 1.13 (1.02-1.25) | 5.9 (4.9-7.0) | 65% |  |
| Machinery mechanics and fitters^1^ | 351 | 32 134 | 1 140 (1 031-1 259) | 1.02 (0.91-1.13) | 14.9 (12.5-17.7) | 42.8% | 106 | 349 (285-416) | | 0.89 (0.73-1.08) | 4.7 (3.3-6.3) | 55% |  |
| Assemblers^1^ | 207 | 20 171 | 1 133 (979-1 285) | 1.02 (0.89-1.17) | 16.1 (12.7-19.5) | 46.8% | 75 | 422 (324-515) | | 1.09 (0.85-1.36) | 6.3 (4.2-8.7) | 67% |  |
| Metal moulders. welders and sheet-metal workers^1^ | 217 | 19 958 | 1 127 (991-1 295) | 1.01 (0.88-1.16) | 14.8 (11.6-18.4) | 42.3% | 67 | 350 (269-443) | | 0.91 (0.71-1.16) | 4.6 (2.9-6.8) | 54% |  |
| Total working population | 10 716 | 963 250 | 1 113 (1 092-1 133) | 1.00 (0.98-1.02) | 13.8 (13.4-14.3) | 38.2% | 3695 | 384 (371-397) | | 1.00 (0.97-1.03) | 4.9 (4.6-5.1) | 57% |  |
| Office clerks | 219 | 20 220 | 1 113 (975-1 260) | 0.99 (0.86-1.13) | 14.2 (11.2-17.2) | 39.9% | 82 | 418 (333-517) | | 1.08 (0.86-1.34) | 5.1 (3.4-7.4) | 59% |  |
| Metal- and mineral-products machine operators^1^ | 288 | 28 152 | 1 112 (990-1 245) | 0.99 (0.88-1.11) | 17.0 (13.7-20.4) | 49.7% | 107 | 417 (340-501) | | 1.08 (0.88-1.30) | 6.6 (4.5-9.3) | 68% |  |
| Agricultural and other mobile-plant operators^1^ | 298 | 28 299 | 1 110 (985-1 243) | 1.00 (0.89-1.12) | 15.9 (13.3-18.7) | 46.2% | 92 | 345 (278-423) | | 0.90 (0.72-1.10) | 4.0 (2.7-5.6) | 48% |  |
| Construction workers^1^ | 483 | 44 639 | 1 106 (1 013-1 209) | 1.00 (0.91-1.09) | 14.9 (12.9-17.0) | 42.8% | 161 | 370 (313-429) | | 0.97 (0.82-1.13) | 5.5 (4.3-7.0) | 62% |  |
| Electricians. tele and electronics repairers^1^ | 171 | 18 399 | 1 047 (890-1 219) | 0.90 (0.77-1.04) | 11.8 (9.1-14.6) | 27.6% | 58 | 372 (271-478) | | 0.90 (0.68-1.16) | 3.6 (2.1-5.1) | 41% |  |
| Accountants | 159 | 15 141 | 1 036 (885-1 202) | 0.93 (0.79-1.08) | 12.7 (9.6-16.3) | 32.8% | 57 | 369 (282-470) | | 0.96 (0.73-1.25) | 5.0 (3.0-7.8) | 58% |  |
| Retail salespersons | 251 | 29 154 | 940 (832-1 064) | 0.84 (0.74-0.95) | 15.2 (12.5-18.2) | 43.8% | 96 | 360 (288-435) | | 0.94 (0.76-1.15) | 5.8 (4.1-7.9) | 64% |  |
| Public service administrative professionals | 181 | 18 347 | 924 (798-1 070) | 0.83 (0.71-0.96) | 14.0 (11.1-17.1) | 39.1% | 71 | 362 (287-455) | | 0.94 (0.74-1.19) | 6.1 (4.1-8.6) | 66% |  |
| Engineers and technicians | 639 | 69 073 | 894 (827-964) | 0.80 (0.74-0.87) | 12.0 (10.6-13.6) | 29.1% | 219 | 307 (263-348) | | 0.80 (0.69-0.91) | 3.8 (2.9-4.8) | 45% |  |
| Senior high school teachers | 148 | 15 718 | 884 (738-1 028) | 0.79 (0.66-0.92) | 10.3 (7.7-13.0) | 17.5% | 47 | 277 (202-360) | | 0.72 (0.53-0.95) | 3.4 (2.0-5.3) | 39% |  |
| Managers of small enterprises | 439 | 49 292 | 881 (797-958) | 0.79 (0.72-0.87) | 11.4 (9.9-13.2) | 25.2% | 134 | 269 (228-314) | | 0.70 (0.59-0.83) | 3.3 (2.4-4.4) | 37% |  |
| Computer technicians and data operators | 82 | 12 342 | 843 (638-1 072) | 0.74 (0.59-0.91) | 11.3 (8.1-15.3) | 24.6% | 31 | 312 (199-451) | | 0.83 (0.57-1.18) | 4.5 (2.6-7.0) | 54% |  |
| Finance and sales associate professionals | 540 | 63 547 | 836 (764-908) | 0.75 (0.69-0.82) | 11.6 (10.1-13.3) | 26.6% | 160 | 247 (209-289) | | 0.65 (0.55-0.75) | 3.3 (2.5-4.2) | 36% |  |
| Computer scientists | 134 | 19 586 | 830 (690-990) | 0.72 (0.60-0.85) | 10.1 (7.4-13.0) | 15.1% | 33 | 203 (135-288) | | 0.53 (0.36-0.74) | 2.9 (1.4-5.1) | 28% |  |
| Elementary school teacher | 93 | 11 065 | 829 (660-1 011) | 0.74 (0.60-0.91) | 11.0 (7.8-14.4) | 22.2% | 31 | 280 (184-388) | | 0.72 (0.49-1.02) | 4.5 (2.5-7.2) | 54% |  |
| College and university teachers | 53 | 6 219 | 795 (593-1 034) | 0.70 (0.53-0.92) | 9.4 (5.5-14.3) | 9.2% | 11 | 160 (72-258) | | 0.42 (0.21-0.75) | 2.9 (0.8-6.8) | 27% |  |
| Directors and chief executives | 121 | 15 716 | 751 (618-898) | 0.68 (0.56-0.81) | 10.8 (8.0-14.2) | 21.3% | 36 | 225 (159-301) | | 0.58 (0.41-0.81) | 2.1 (1.0-3.5) | 1% |  |
| Business professionals | 195 | 25 713 | 740 (636-853) | 0.66 (0.57-0.76) | 9.3 (7.3-11.4) | 7.8% | 72 | 276 (216-338) | | 0.71 (0.55-0.89) | 3.1 (2.0-4.5) | 33% |  |
| Production and operations managers | 226 | 30 474 | 739 (644-846) | 0.66 (0.58-0.75) | 9.0 (7.2-11.1) | 4.7% | 59 | 195 (146-254) | | 0.50 (0.38-0.65) | 2.1 (1.3-3.3) | 0% |  |
| Architects and civil engineers | 165 | 22 282 | 728 (623-845) | 0.65 (0.56-0.76) | 9.5 (7.2-12.0) | 9.8% | 56 | 249 (183-321) | | 0.64 (0.49-0.83) | 3.0 (1.6-4.7) | 30% |  |
| Specialist managers | 176 | 27 888 | 633 (544-732) | 0.57 (0.49-0.66) | 8.5 (6.7-10.8) | 0.0% | 66 | 240 (186-299) | | 0.62 (0.48-0.79) | 3.2 (2.1-4.6) | 35% |  |
| ^1^Occupations classified as being in the lowest socio-economic status group. i.e. “skilled and unskilled manual workers” according to Statistics Sweden. ^b^Personal carers includes child-care workers, assistant nurses, hospital ward assistants, home-based personal care, attendants in psychiatric care and dental nurses. | | | | | | | | | | | | | |

**Table S5. ischemic heart disease (IHD) and stroke 2002-2015 in men with type 2 diabetes across the 30 most common occupations in Swedish men.**

|  |  | | | IHD | | | | | | STROKE | | | | | |
| --- | --- | --- | --- | --- | --- | --- | --- | --- | --- | --- | --- | --- | --- | --- | --- |
|  | | **No. Cases** | **Person-years** | | **Age stand Incidence per 100 000 (95% CI)** | **SIR**  **(95% CI)** | **Absolute**  **10-year risk at age 60** | **AR%** | **No. Cases** | | **Age stand Incidence per 100 000**  **(95% CI)** | **SIR**  **(95% CI)** | **Absolute**  **10-year risk at age 60** | **AR%** |  |
| Manufacturing labourers^1^ | | 668 | 23 615 | | 2 841 (2 528-3 159) | 1.28 (1.19-1.38) | 24.7 (20.4-29.3) | 40.8% | 171 | | 753 (630-889) | 1.21 (1.04-1.41) | 9.6 (7.0-12.7) | 58.5% |  |
| Metal moulders. welders and sheet-metal workers^1^ | | 497 | 19 958 | | 2 536 (2 239-2 883) | 1.15 (1.05-1.25) | 22.6 (18.2-27.8) | 35.3% | 115 | | 597 (485-721) | 0.96 (0.80-1.16) | 6.1 (4.2-8.2) | 35.0% |  |
| Motor-vehicle drivers^1^ | | 2 241 | 95 437 | | 2 462 (2 315-2 606) | 1.11 (1.06-1.15) | 22.9 (21.0-24.8) | 36.1% | 681 | | 771 (705-836) | 1.24 (1.15-1.34) | 9.6 (8.3-11.0) | 58.6% |  |
| Office clerks | | 488 | 20 220 | | 2 440 (2 110-2 766) | 1.10 (1.00-1.20) | 19.8 (15.9-24.1) | 26.1% | 128 | | 640 (515-775) | 1.04 (0.87-1.23) | 8.0 (5.5-10.8) | 50.4% |  |
| Metal- and mineral-products machine operators^1^ | | 648 | 28 152 | | 2 431 (2 187-2 698) | 1.09 (1.01-1.18) | 23.7 (20.0-27.6) | 38.4% | 163 | | 625 (521-734) | 1.01 (0.86-1.17) | 7.7 (5.5-10.2) | 48.3% |  |
| Assemblers^1^ | | 453 | 20 171 | | 2 424 (2 093-2 785) | 1.09 (0.99-1.19) | 21.4 (17.4-25.8) | 31.8% | 131 | | 701 (542-877) | 1.16 (0.97-1.38) | 9.8 (6.7-13.3) | 59.7% |  |
| Personal care takers^1b^ | | 729 | 35 768 | | 2 413 (2 152-2 663) | 1.08 (1.00-1.16) | 22.0 (18.7-25.1) | 33.5% | 186 | | 699 (570-840) | 1.06 (0.91-1.22) | 7.2 (5.5-9.1) | 44.8% |  |
| Building finishers and related trades workers^1^ | | 1 403 | 59 659 | | 2 372 (2 191-2 572) | 1.07 (1.01-1.13) | 21.4 (19.2-23.8) | 31.8% | 386 | | 655 (583-738) | 1.06 (0.96-1.17) | 8.5 (7.0-10.2) | 53.0% |  |
| Stores and transport clerks | | 755 | 33 455 | | 2 357 (2 092-2 675) | 1.06 (0.98-1.14) | 21.7 (18.4-25.6) | 32.5% | 188 | | 597 (507-691) | 0.96 (0.83-1.11) | 7.7 (5.8-9.8) | 48.6% |  |
| Machine operators and assemblers^1^ | | 245 | 12 124 | | 2 341 (1 943-2 789) | 1.01 (0.89-1.15) | 24.3 (19.0-30.6) | 39.8% | 74 | | 702 (533-897) | 1.14 (0.90-1.44) | 10.8 (7.2-15.0) | 63.1% |  |
| Machinery mechanics and fitters^1^ | | 719 | 32 134 | | 2 282 (2 060-2 524) | 1.03 (0.95-1.10) | 24.3 (20.9-27.6) | 39.8% | 172 | | 554 (463-652) | 0.89 (0.76-1.04) | 7.2 (5.3-9.5) | 45.0% |  |
| Agricultural and other mobile-plant operators^1^ | | 621 | 28 299 | | 2 282 (2 030-2 544) | 1.02 (0.94-1.10) | 20.0 (16.6-23.5) | 27.0% | 184 | | 676 (565-787) | 1.10 (0.95-1.27) | 9.8 (7.4-12.5) | 59.5% |  |
| Total working population | | 21 372 | 963 250 | | 2 219 (2 174-2 262) | 1.00 (0.99-1.01) | 19.7 (19.1-20.3) | 25.8% | 5968 | | 620 (602-637) | 1.00 (0.98-1.03) | 7.3 (7.0-7.7) | 45.8% |  |
| Construction workers^1^ | | 960 | 44 639 | | 2 202 (1 990-2 416) | 0.98 (0.92-1.05) | 19.2 (16.9-21.8) | 24.0% | 242 | | 555 (478-636) | 0.90 (0.79-1.02) | 6.8 (5.3-8.5) | 41.9% |  |
| Retail salespersons | | 611 | 29 154 | | 2 199 (1 905-2 553) | 1.01 (0.93-1.09) | 23.4 (19.2-28.6) | 37.5% | 146 | | 542 (446-642) | 0.88 (0.75-1.04) | 6.0 (4.2-8.2) | 33.8% |  |
| Managers of small enterprises | | 1 038 | 49 292 | | 2 072 (1 891-2 280) | 0.93 (0.88-0.99) | 19.1 (16.7-21.9) | 23.6% | 317 | | 633 (554-714) | 1.02 (0.91-1.14) | 7.3 (5.7-8.9) | 45.5% |  |
| Accountants | | 314 | 15 141 | | 2 037 (1 733-2 358) | 0.91 (0.82-1.02) | 17.9 (14.2-22.2) | 18.3% | 86 | | 547 (428-672) | 0.90 (0.72-1.11) | 8.5 (5.7-11.9) | 53.5% |  |
| Electricians. tele and electronics repairers^1^ | | 348 | 18 399 | | 1 993 (1 671-2 333) | 0.89 (0.79-0.98) | 17.8 (14.2-21.7) | 17.7% | 95 | | 560 (435-686) | 0.89 (0.72-1.09) | 7.1 (4.8-9.6) | 44.3% |  |
| Finance and sales associate professionals | | 1 268 | 63 547 | | 1 958 (1 794-2 144) | 0.88 (0.84-0.93) | 17.4 (15.3-19.6) | 15.9% | 339 | | 525 (465-593) | 0.84 (0.76-0.94) | 6.1 (5.0-7.5) | 35.2% |  |
| Engineers and technicians | | 1 398 | 69 073 | | 1 956 (1 822-2 111) | 0.89 (0.84-0.93) | 18.0 (16.1-20.0) | 19.0% | 341 | | 478 (423-539) | 0.77 (0.69-0.85) | 5.4 (4.4-6.4) | 26.1% |  |
| Architects and civil engineers | | 439 | 22 282 | | 1 952 (1 709-2 220) | 0.88 (0.80-0.97) | 18.1 (14.8-21.6) | 19.2% | 105 | | 475 (377-584) | 0.75 (0.61-0.90) | 4.0 (2.7-5.6) | 0.0% |  |
| Business professionals | | 508 | 25 713 | | 1 940 (1 643-2 358) | 0.87 (0.79-0.95) | 15.9 (12.9-19.5) | 8.2% | 125 | | 480 (383-579) | 0.76 (0.63-0.90) | 4.3 (3.0-5.8) | 8.1% |  |
| Public service administrative professionals | | 366 | 18 347 | | 1 911 (1 627-2 210) | 0.86 (0.77-0.95) | 18.5 (14.3-22.9) | 20.8% | 120 | | 615 (478-769) | 0.99 (0.82-1.18) | 7.8 (5.4-10.7) | 49.0% |  |
| Elementary school teacher | | 213 | 11 065 | | 1 904 (1 546-2 254) | 0.85 (0.74-0.98) | 17.9 (13.6-23.0) | 18.3% | 58 | | 518 (381-680) | 0.83 (0.63-1.08) | 6.3 (3.7-9.9) | 36.9% |  |
| Computer scientists | | 320 | 19 586 | | 1 834 (1 527-2 175) | 0.81 (0.72-0.90) | 16.5 (13.1-20.2) | 11.5% | 88 | | 545 (406-699) | 0.85 (0.68-1.05) | 7.3 (4.7-10.8) | 45.8% |  |
| Computer technicians and data operators | | 178 | 12 342 | | 1 833 (1 408-2 290) | 0.75 (0.64-0.87) | 14.6 (10.2-19.7) | 0.0% | 40 | | 447 (316-632) | 0.65 (0.46-0.88) | 4.5 (2.5-7.2) | 11.6% |  |
| Senior high school teachers | | 300 | 15 718 | | 1 805 (1 552-2 062) | 0.82 (0.73-0.92) | 17.3 (13.3-21.5) | 15.5% | 91 | | 530 (397-672) | 0.87 (0.70-1.06) | 7.2 (4.8-9.9) | 44.7% |  |
| Production and operations managers | | 547 | 30 474 | | 1 762 (1 536-1 973) | 0.80 (0.73-0.87) | 16.8 (13.9-20.0) | 13.2% | 166 | | 533 (439-635) | 0.87 (0.74-1.01) | 6.8 (5.1-8.7) | 41.3% |  |
| Directors and chief executives | | 278 | 15 716 | | 1 715 (1 426-2 016) | 0.77 (0.69-0.87) | 17.3 (13.0-22.4) | 15.7% | 95 | | 593 (468-718) | 0.95 (0.77-1.16) | 6.6 (4.5-8.9) | 39.9% |  |
| Specialist managers | | 482 | 27 888 | | 1 708 (1 511-1 926) | 0.77 (0.70-0.84) | 15.6 (12.7-18.9) | 6.3% | 135 | | 486 (392-590) | 0.78 (0.65-0.92) | 5.5 (3.8-7.6) | 27.2% |  |
| College and university teachers | | 111 | 6 219 | | 1 653 (1 223-2 065) | 0.76 (0.63-0.92) | 16.9 (10.9-25.4) | 13.7% | 32 | | 453 (299-622) | 0.77 (0.52-1.08) | 7.1 (3.5-12.2) | 44.2% |  |
| ^1^ Occupations classified as being in the lowest socio-economic status group. i.e. “skilled and unskilled manual workers” according to Statistics Sweden. ^b^Personal carers includes child-care workers, assistant nurses, hospital ward assistants, home-based personal care, attendants in psychiatric care and dental nurses. | | | | | | | | | | | | | | | |

**Table S6. all-cause and CVD mortality 2002-2015 in women with type 2 diabetes across the 30 most common occupations in Swedish women.**

|  | All-cause mortality | | | | | | | CVD mortality | | | | | |  |
| --- | --- | --- | --- | --- | --- | --- | --- | --- | --- | --- | --- | --- | --- | --- |
|  | **No. Cases** | **Person-years** | **Age stand per**  **100 000 (95% CI)** | **SIR**  **(95% CI)** | **Absolute**  **10-year risk at age 60** | **AR%** | **No. Cases** | | **Age stand per 100 000**  **(95% CI)** | **SIR**  **(95% CI)** | **Absolute**  **10-year risk at age 60** | **AR%** |  |  |
| Manufacturing labourers^1^ | 144 | 12295 | 1 150 (966-1 354) | 1.53 (1.29-1.80) | 12.4 (8.9-16.5) | 65.5% | 51 | | 392 (287-512) | 2.31 (1.72-3.04) | 3.3 (1.7-5.3) | 76.0% |  |  |
| Cleaners^1^ | 282 | 33476 | 876 (778-972) | 1.13 (1.00-1.27) | 10.2 (8.2-12.2) | 58.1% | 72 | | 225 (175-281) | 1.26 (0.98-1.58) | 2.6 (1.6-3.8) | 69.4% |  |  |
| Client information clerks | 92 | 11605 | 817 (656-1 006) | 1.06 (0.85-1.30) | 10.5 (7.2-14.5) | 59.5% | 16 | | 144 (76-216) | 0.81 (0.46-1.31) | 1.5 (0.6-3.1) | 48.2% |  |  |
| Cooks, waitresses and housekeepers^1^ | 122 | 15263 | 789 (649-931) | 1.01 (0.84-1.21) | 11.6 (8.4-15.3) | 63.3% | 36 | | 230 (153-310) | 1.28 (0.89-1.77) | 5.0 (2.7-8.5) | 84.2% |  |  |
| Total working population | 4165 | 540199 | 771 (748-793) | 1.00 (0.97-1.03) | 10.0 (9.6-10.6) | 57.6% | 966 | | 179 (167-190) | 1.00 (0.94-1.07) | 2.4 (2.1-2.7) | 66.9% |  |  |
| Accountants | 154 | 22179 | 675 (570-787) | 0.88 (0.75-1.04) | 10.8 (8.3-13.7) | 60.7% | 31 | | 137 (90-191) | 0.78 (0.53-1.10) | 2.4 (1.4-3.7) | 67.0% |  |  |
| Office clerks | 320 | 39872 | 769 (690-860) | 1.00 (0.89-1.11) | 11.9 (10.0-14.2) | 64.1% | 63 | | 150 (113-189) | 0.84 (0.65-1.07) | 1.9 (1.1-2.9) | 57.9% |  |  |
| Personal carers^1b^ | 1211 | 170608 | 765 (723-810) | 0.99 (0.93-1.04) | 10.5 (9.6-11.5) | 59.5% | 288 | | 187 (163-208) | 1.04 (0.92-1.16) | 2.8 (2.3-3.4) | 72.0% |  |  |
| Retail salespersons^1^ | 199 | 25982 | 753 (653-863) | 0.99 (0.86-1.14) | 9.8 (7.7-12.4) | 56.4% | 35 | | 131 (91-176) | 0.75 (0.52-1.04) | 1.4 (0.7-2.6) | 45.3% |  |  |
| Kitchen assistants^1^ | 175 | 23460 | 752 (644-875) | 0.97 (0.83-1.12) | 11.6 (9.0-14.8) | 63.4% | 39 | | 164 (114-221) | 0.93 (0.66-1.26) | 2.2 (1.0-4.1) | 64.1% |  |  |
| Psychologists and social workers | 59 | 8139 | 727 (546-922) | 0.94 (0.71-1.21) | 6.2 (3.7-9.6) | 31.6% | 14 | | 172 (84-272) | 0.97 (0.53-1.62) | 1.1 (0.3-2.9) | 30.5% |  |  |
| Office secretaries and data entry operators | 117 | 16202 | 716 (595-845) | 0.92 (0.76-1.10) | 10.0 (7.2-13.2) | 57.4% | 32 | | 194 (133-257) | 1.09 (0.74-1.53) | 2.9 (1.6-4.8) | 73.0% |  |  |
| Finance and sales associate professionals | 86 | 13799 | 678 (530-817) | 0.86 (0.69-1.06) | 7.5 (4.9-10.3) | 43.4% | 16 | | 142 (78-225) | 0.71 (0.40-1.15) | 1.6 (0.4-3.8) | 50.5% |  |  |
| Social work associate professionals^c^ | 49 | 8150 | 675 (489-892) | 0.84 (0.62-1.11) | 8.1 (4.9-12.5) | 47.5% | 9 | | 124 (49-212) | 0.69 (0.32-1.31) | 2.2 (0.4-6.0) | 64.0% |  |  |
| Senior high school teachers | 58 | 8492 | 653 (508-833) | 0.85 (0.65-1.10) | 8.5 (5.2-12.9) | 50.1% | 15 | | 168 (90-265) | 0.94 (0.52-1.54) | 1.5 (0.4-3.3) | 48.7% |  |  |
| Numerical clerks | 170 | 24750 | 648 (558-749) | 0.85 (0.72-0.98) | 11.2 (8.7-14.1) | 61.9% | 32 | | 120 (80-164) | 0.68 (0.47-0.96) | 2.1 (1.0-3.7) | 62.5% |  |  |
| Nurses | 92 | 14663 | 640 (513-770) | 0.85 (0.68-1.04) | 9.1 (6.3-12.4) | 53.0% | 19 | | 134 (73-196) | 0.77 (0.46-1.20) | 2.5 (0.9-5.3) | 67.9% |  |  |
| Computer scientists | 15 | 3212 | 626 (296-1 103) | 0.81 (0.45-1.33) | 10.7 (3.8-20.2) | 60.1% | 4 | | 215 (-) | 1.05 (0.29-2.68) | 4.0 (0.0-11.5) | 80.3% |  |  |
| Engineers and technicians | 29 | 5345 | 604 (389-855) | 0.75 (0.51-1.08) | 6.8 (2.9-12.5) | 37.7% | 6 | | 119 (-) | 0.69 (0.25-1.51) | 1.4 (-) | 44.1% |  |  |
| Production and operations managers | 48 | 8857 | 602 (428-825) | 0.73 (0.54-0.97) | 7.4 (4.6-11.4) | 42.4% | 7 | | 84 (26-166) | 0.48 (0.19-0.99) | 1.1 (0.1-3.6) | 30.1% |  |  |
| Cashiers. tellers and related clerks | 41 | 6659 | 601 (440-788) | 0.77 (0.55-1.04) | 7.0 (3.5-11.6) | 39.2% | 10 | | 143 (63-252) | 0.79 (0.38-1.46) | 1.2 (0.2-4.1) | 35.7% |  |  |
| Specialist managers | 21 | 4244 | 594 (361-889) | 0.72 (0.44-1.09) | 6.7 (2.8-12.3) | 36.4% | 7 | | 199 (68-381) | 1.10 (0.44-2.26) | 2.5 (0.3-3.2) | 46.7% |  |  |
| Business professionals | 73 | 12487 | 590 (452-742) | 0.78 (0.61-0.98) | 9.2 (6.3-12.6) | 53.5% | 16 | | 149 (78-238) | 0.76 (0.43-1.23) | 2.2 (0.9-4.3) | 64.4% |  |  |
| Public service administrative professionals | 64 | 10863 | 588 (447-747) | 0.78 (0.60-1.00) | 9.4 (6.0-13.7) | 54.8% | 16 | | 157 (84-256) | 0.86 (0.49-1.40) | 3.1 (1.1-6.9) | 74.8% |  |  |
| Physiotherapists and dental hygienists | 29 | 5195 | 587 (403-839) | 0.76 (0.51-1.09) | 6.4 (3.2-10.6) | 33.1% | 5 | | 105 (-) | 0.57 (0.19-1.34) | 1.0 (-) | 20.8% |  |  |
| Preschool and recreation teachers | 80 | 15305 | 582 (466-719) | 0.76 (0.60-0.94) | 6.6 (4.3-9.1) | 35.3% | 14 | | 109 (58-174) | 0.60 (0.33-1.01) | 1.4 (0.4-3.1) | 45.5% |  |  |
| Midwifes and specialist nurses | 50 | 9963 | 509 (366-659) | 0.67 (0.50-0.88) | 8.1 (5.1-11.4) | 47.3% | 6 | | 65 (-) | 0.35 (0.13-0.76) | 1.1 (-) | 31.2% |  |  |
| Elementary school teacher | 72 | 14892 | 493 (385-607) | 0.63 (0.50-0.80) | 5.8 (3.6-8.6) | 26.1% | 12 | | 81 (39-130) | 0.46 (0.24-0.80) | 2.1 (0.6-4.6) | 62.0% |  |  |
| Managers of small enterprises | 46 | 9670 | 468 (348-606) | 0.61 (0.45-0.82) | 8.2 (5.1-12.4) | 48.2% | 10 | | 106 (44-179) | 0.58 (0.28-1.06) | 2.0 (0.2-5.2) | 60.7% |  |  |
| Health professionals | 17 | 4958 | 461 (264-705) | 0.53 (0.31-0.86) | 4.7 (1.8-9.9) | 10.1% | 2 | | 57 (8-149) | 0.29 (0.04-1.05) | 0.2 (0.0-1.1) | - |  |  |
| Writers and creative or performing artists | 14 | 3269 | 458 (245-728) | 0.59 (0.32-0.99) | 4.3 (1.3-8.6) | 0.0% | 4 | | 135 (-) | 0.74 (0.20-1.89) | 0.8 (-) | 0.0% |  |  |
| ^1^ Occupations classified as being in the lowest socio-economic status group. i.e. “skilled and unskilled manual workers” according to Statistics Sweden. ^b^Personal carers includes child-care workers, assistant nurses, hospital ward assistants, home-based personal care, attendants in psychiatric care and dental nurses. ^c^Social work associate professionals includes treatment assistants and youth recreations leaders. For some occupations, confidence intervals could not be estimated as the number of cases was too small. | | | | | | | | | | | | | | |

**Table S7. ischemic heart disease (IHD) and stroke 2002-2005 in women with type 2 diabetes across the 30 most common occupations in Swedish women.**

|  | IHD | | |  |  | | |  | | STROKE |  |  |  |  |
| --- | --- | --- | --- | --- | --- | --- | --- | --- | --- | --- | --- | --- | --- | --- |
|  | No. Cases | Person-years | Age stand Incidence per 100 000  (95% CI) | SIR  (95% CI) | Absolute  10-year risk at age 60 | AR% | No. Cases | | Age stand Incidence per 100 000  (95% CI) | | SIR  (95% CI) | Absolute  10-year risk at age 60 | AR% |  |
| Manufacturing labourers^1^ | 162 | 12 295 | 1 262 (991-1 574) | 1.26 (1.07-1.47) | 11.5 (7.7-16.2) | 52.3% | 69 | | 534 (405-689) | | 1.54 (1.20-1.95) | 4.6 (2.5-7.3) | 72.3% |  |
| Social work associate professionals^c^ | 98 | 8 150 | 1 314 (922-1 750) | 1.29 (1.05-1.57) | 11.1 (5.5-18.5) | 51.0% | 27 | | 357 (218-512) | | 1.00 (0.66-1.46) | 3.4 (1.5-6.2) | 64.7% |  |
| Cashiers. tellers and related clerks^1^ | 86 | 6 659 | 1 262 (916-1 624) | 1.23 (0.98-1.52) | 10.9 (6.4-16.5) | 49.9% | 27 | | 388 (216-594) | | 1.07 (0.70-1.55) | 2.8 (1.0-5.9) | 58.3% |  |
| Health professionals | 44 | 4 958 | 1206 (568-2 030) | 1.09 (0.79-1.46) | 7.6 (1.9-16.6) | 28.0% | 3 | | 87 (-) | | 0.21 (0.04-0.60) | 1.6 (0.3-4.3) | 31.0% |  |
| Psychologists and social workers | 96 | 8 139 | 1 160 (741-1 681) | 1.17 (0.95-1.43) | 10.2 (5.2-17.5) | 46.6% | 41 | | 504 (330-692) | | 1.40 (1.00-1.90) | 3.7 (1.7-6.5) | 67.1% |  |
| Retail salespersons^1^ | 296 | 25 982 | 1 156 (914-1 513) | 1.15 (1.03-1.29) | 12.6 (8.0-18.4) | 56.5% | 96 | | 365 (284-451) | | 1.02 (0.83-1.24) | 4.7 (3.1-6.7) | 72.7% |  |
| Managers of small enterprises | 107 | 9 670 | 1 127 (797-1 455) | 1.10 (0.90-1.33) | 6.2 (3.7-9.1) | 11.4% | 30 | | 309 (214-429) | | 0.86 (0.58-1.22) | 3.7 (1.7-6.6) | 66.6% |  |
| Personal carers^1b^ | 1802 | 170 608 | 1 118 (1 043-1 190) | 1.11 (1.06-1.17) | 9.3 (8.3-10.3) | 41.4% | 624 | | 394 (358-429) | | 1.09 (1.01-1.18) | 4.9 (4.1-5.5) | 73.5% |  |
| Kitchen assistants^1^ | 257 | 23 460 | 1 094 (828-1 514) | 1.10 (0.97-1.24) | 11.0 (7.6-15.9) | 50.4% | 81 | | 345 (272-423) | | 0.95 (0.76-1.19) | 3.9 (2.5-5.7) | 68.4% |  |
| Office clerks | 422 | 39 872 | 1 019 (880-1 167) | 1.02 (0.92-1.12) | 9.4 (7.5-12.0) | 42.2% | 154 | | 374 (308-449) | | 1.03 (0.87-1.20) | 4.1 (3.1-5.3) | 69.4% |  |
| Cleaners^1^ | 331 | 33 476 | 1 008 (840-1 186) | 1.02 (0.91-1.13) | 7.6 (5.3-10.3) | 28.3% | 127 | | 394 (318-473) | | 1.09 (0.91-1.30) | 4.8 (3.3-6.7) | 73.2% |  |
| Total working population | 5391 | 540 199 | 998 (959-1 038) | 1.00 (0.97-1.03) | 8.8 (8.2-9.4) | 37.8% | 1949 | | 361 (343-380) | | 1.00 (0.96-1.05) | 4.1 (3.8-4.5) | 69.9% |  |
| Office secretaries and data entry operators | 166 | 16 202 | 993 (790-1 217) | 1.00 (0.85-1.16) | 8.8 (6.1-11.7) | 37.7% | 40 | | 244 (165-327) | | 0.67 (0.48-0.92) | 3.2 (1.7-5.4) | 63.0% |  |
| Cooks. waitresses and housekeepers^1^ | 143 | 15 263 | 935 (725-1 153) | 0.91 (0.77-1.07) | 12.7 (8.4-17.8) | 56.9% | 57 | | 360 (267-473) | | 1.01 (0.76-1.30) | 3.2 (1.7-5.6) | 62.9% |  |
| Writers and creative or performing artists | 28 | 3 269 | 930 (431-1 491) | 0.91 (0.60-1.31) | 6.0 (1.3-12.5) | 8.7% | 7 | | 227 (75-407) | | 0.63 (0.26-1.31) | 8.7 (1.9-20.2) | 82.5% |  |
| Preschool and recreation teachers | 122 | 15 305 | 915 (713-1 144) | 0.87 (0.73-1.04) | 8.3 (6.0-11.1) | 34.1% | 46 | | 369 (239-513) | | 0.95 (0.69-1.26) | 2.7 (1.3-4.8) | 57.1% |  |
| Client information clerks | 102 | 11 605 | 910 (667-1 150) | 0.91 (0.74-1.10) | 6.0 (3.3-9.5) | 9.3% | 48 | | 424 (291-548) | | 1.19 (0.88-1.58) | 5.3 (2.9-8.0) | 75.1% |  |
| Midwifes and specialist nurses | 85 | 9 963 | 851 (627-1 120) | 0.86 (0.69-1.06) | 6.0 (3.7-8.9) | 8.9% | 26 | | 264 (167-382) | | 0.74 (0.49-1.09) | 4.3 (2.1-7.2) | 70.7% |  |
| Nurses | 119 | 14 663 | 849 (612-1 091) | 0.84 (0.70-1.01) | 6.8 (4.3-9.7) | 19.9% | 43 | | 313 (219-420) | | 0.85 (0.62-1.15) | 3.0 (1.6-4.9) | 61.0% |  |
| Numerical clerks | 211 | 24 750 | 819 (668-992) | 0.82 (0.71-0.93) | 8.0 (5.7-11.2) | 32.0% | 75 | | 289 (212-374) | | 0.80 (0.63-1.00) | 3.7 (2.4-5.4) | 66.9% |  |
| Accountants | 175 | 22 179 | 803 (645-985) | 0.79 (0.67-0.91) | 6.6 (4.5-8.8) | 16.6% | 71 | | 325 (239-431) | | 0.88 (0.69-1.11) | 3.5 (2.2-5.2) | 65.7% |  |
| Public service administrative professionals | 73 | 10 863 | 732 (521-951) | 0.69 (0.54-0.87) | 7.0 (3.4-11.3) | 21.7% | 25 | | 235 (136-342) | | 0.66 (0.43-0.98) | 3.8 (1.6-6.9) | 67.7% |  |
| Senior high school teachers | 61 | 8 492 | 703 (483-955) | 0.70 (0.53-0.90) | 8.1 (4.5-12.7) | 32.7% | 19 | | 215 (108-370) | | 0.59 (0.36-0.93) | 2.5 (0.7-5.7) | 54.5% |  |
| Finance and sales associate professionals | 91 | 13 799 | 697 (499-937) | 0.69 (0.56-0.85) | 6.0 (3.3-9.4) | 8.3% | 46 | | 366 (249-482) | | 0.99 (0.72-1.32) | 3.8 (2.2-6.1) | 67.7% |  |
| Specialist managers | 26 | 4 244 | 683 (356-1 137) | 0.67 (0.44-0.98) | 7.2 (2.6-12.5) | 23.6% | 13 | | 349 (182-624) | | 0.97 (0.52-1.65) | 4.4 (1.3-9.7) | 71.3% |  |
| Elementary school teacher | 97 | 14 892 | 660 (501-829) | 0.66 (0.54-0.81) | 6.0 (3.7-8.8) | 8.8% | 52 | | 355 (246-481) | | 0.98 (0.73-1.29) | 4.0 (2.0-6.4) | 68.7% |  |
| Production and operations managers | 53 | 8 857 | 643 (412-911) | 0.63 (0.48-0.83) | 5.5 (2.9-8.9) | 0.0% | 19 | | 214 (124-318) | | 0.63 (0.38-0.99) | 2.5 (0.8-4.9) | 53.2% |  |
| Computer scientists | 19 | 3 212 | 634 (254-1 148) | 0.76 (0.46-1.19) | 7.6 (1.7-13.3) | 28.0% | 4 | | - | | 0.48 (0.13-1.22) | - | 69.1% |  |
| Business professionals | 73 | 12 487 | 618 (423-840) | 0.61 (0.48-0.76) | 6.4 (3.5-11.0) | 14.2% | 25 | | 227 (134-332) | | 0.58 (0.38-0.86) | 2.1 (1.0-3.5) | 45.6% |  |
| Physiotherapists and dental hygienists | 29 | 5 195 | 586 (323-881) | 0.59 (0.39-0.84) | 6.5 (2.7-11.3) | 15.9% | 8 | | 169 (55-328) | | 0.45 (0.20-0.89) | 1.1 (0.1-3.0) | 0.0% |  |
| Engineers and technicians | 26 | 5 345 | 501 (284-745) | 0.52 (0.34-0.76) | 7.8 (2.7-14.7) | 29.9% | 19 | | 378 (214-557) | | 1.07 (0.64-1.66) | 5.6 (2.0-11.3) | 76.2% |  |
| ^1^ Occupations classified as being in the lowest socio-economic status group. i.e. “skilled and unskilled manual workers” according to Statistics Sweden. ^b^Personal carers includes child-care workers, assistant nurses, hospital ward assistants, home-based personal care, attendants in psychiatric care and dental nurses. ^c^Social work associate professionals includes treatment assistants and youth recreations leaders. For some occupations, confidence intervals could not be estimated as the number of cases was too small. | | | | | | | | | | | | | | |

Table S8. CVD risk factors in men with TYPE 2 DIABETES across the 30 most common occupational groups in Sweden 2002-2015.

|  | Diabetes duration (years) | HbA_1c_ (mmol/mol) | | LDL-cholesterol (mmol/l) | | Systolic blood pressure (mmhg) | | | eGFR | | Albumi-nuria | BMI (kg/m^2^) | | | Smoking | | Physical activity | |
| --- | --- | --- | --- | --- | --- | --- | --- | --- | --- | --- | --- | --- | --- | --- | --- | --- | --- | --- |
|  | **mean (sd)** | **mean (sd)** | **% above target** | **mean (sd)** | **% above target** | **mean (sd)** | **% above target** | **mean (sd)** | | **%** | | **Mean (sd)** | **% with obesity** | **% smokers** | | **% active**  **<30 min**  **/week** | | |
| Manufacturing labourers | 6.5 (5.3) | 56 (16) | 47.6 | 2.8 (1.0) | 56.4 | 137 (17) | 29.4 | 92.8 (23.2) | | 24.3 | | 30.5 (4.9) | 50.7 | 25.1 | | 23.5 | | |
| Machine operators | 5.8 (5.0) | 55 (14) | 45.5 | 2.7 (0.9) | 56.6 | 135 (15) | 26.7 | 94.4 (22.9) | | 19.3 | | 30.5 (5.0) | 47.9 | 19.4 | | 26.6 | | |
| Personal carers^b^ | 6.1 (5.2) | 55 (15) | 44.2 | 2.8 (0.9) | 57.1 | 134 (15) | 24.6 | 95.5 (23.2) | | 20.3 | | 30.4 (5.1) | 49.3 | 22.5 | | 23.8 | | |
| Stores and transport clerks | 6.0 (5.2) | 55 (15) | 43.0 | 2.7 (0.9) | 57.1 | 135 (15) | 26.3 | 93.8 (21.9) | | 21.7 | | 30.9 (5.2) | 52.1 | 19.3 | | 23.6 | | |
| Building finishers and related trades workers | 6.4 (5.4) | 54 (14) | 41.3 | 2.8 (0.9) | 57.3 | 137 (16) | 29.4 | 93.3 (21.7) | | 21.5 | | 30.5 (4.8) | 49.6 | 20.5 | | 24.9 | | |
| Motor-vehicle drivers | 6.5 (5.2) | 56 (15) | 46.1 | 2.8 (0.9) | 56.6 | 135 (15) | 26.2 | 92.1 (21.1) | | 22.2 | | 31.0 (5.1) | 52.9 | 22.3 | | 30.8 | | |
| Machinery mechanics and fitters | 6.3 (5.3) | 55 (15) | 42.7 | 2.8 (0.9) | 57.5 | 136 (15) | 27.7 | 93.0 (20.8) | | 21.4 | | 30.5 (4.7) | 49.9 | 19.2 | | 30.2 | | |
| Assemblers | 6.1 (5.1) | 55 (15) | 42.6 | 2.8 (0.9) | 57.8 | 134 (15) | 25.7 | 92.9 (21.1) | | 19.7 | | 30.3 (5.0) | 46.3 | 19.4 | | 24.5 | | |
| Metal moulders. welders and sheet-metal workers | 5.9 (4.9) | 54 (14) | 42.0 | 2.8 (0.9) | 56.6 | 136 (15) | 28.2 | 94.1 (21.6) | | 22.1 | | 30.5 (4.7) | 49.5 | 22.4 | | 28.1 | | |
| Office clerks | 6.6 (5.4) | 54 (15) | 42.2 | 2.7 (0.9) | 54.2 | 135 (15) | 26.8 | 90.9 (21.9) | | 20.5 | | 30.7 (5.4) | 49.5 | 14.7 | | 25.5 | | |
| Total working population | 6.4 (5.3) | 54 (15) | 41.9 | 2.8 (0.9) | 56.1 | 136 (15) | 27.1 | 91.7 (21.5) | | 20.9 | | 30.4 (4.9) | 48.1 | 17.9 | | 24.9 | | |
| Metal- and mineral-products machine operators | 6.2 (5.3) | 55 (14) | 44.3 | 2.8 (0.9) | 57.6 | 136 (15) | 26.3 | 93.2 (20.4) | | 19.8 | | 30.8 (5.0) | 52.1 | 21.4 | | 23.8 | | |
| Agricultural and other mobile-plant operators | 6.6 (5.2) | 56 (15) | 47.2 | 2.8 (0.9) | 57.9 | 136 (15) | 27.8 | 92.2 (21.0) | | 22.9 | | 31.6 (5.2) | 58.1 | 19.7 | | 31.5 | | |
| Construction workers | 6.0 (5.0) | 54 (14) | 41.9 | 2.9 (0.9) | 60.4 | 136 (15) | 27.0 | 94.1 (21.6) | | 20.7 | | 30.5 (4.7) | 50.7 | 20.6 | | 23.7 | | |
| Electricians. tele and electronics repairers | 6.1 (5.2) | 54 (14) | 41.2 | 2.8 (0.9) | 57.4 | 136 (15) | 27.8 | 91.7 (20.4) | | 19.9 | | 30.4 (4.8) | 47.8 | 15.8 | | 25.2 | | |
| Accountants | 6.5 (5.1) | 54 (14) | 41.9 | 2.7 (0.9) | 52.8 | 136 (15) | 26.4 | 89.0 (22.3) | | 22.1 | | 30.6 (5.0) | 49.7 | 12.3 | | 24.3 | | |
| Retail salespersons | 6.2 (5.2) | 54 (15) | 40.2 | 2.8 (0.9) | 56.9 | 135 (15) | 27.2 | 93.4 (21.7) | | 20.2 | | 30.2 (4.8) | 46.4 | 18.4 | | 25.3 | | |
| Public service administrative professionals | 6.6 (5.3) | 52 (13) | 36.6 | 2.8 (0.9) | 57.9 | 136 (15) | 28.2 | 87.4 (19.8) | | 20.4 | | 30.0 (4.8) | 45.8 | 11.4 | | 18.5 | | |
| Engineers and technicians | 6.2 (5.2) | 53 (13) | 37.8 | 2.8 (0.9) | 57.3 | 136 (15) | 27.4 | 88.3 (19.7) | | 19.2 | | 30.1 (4.8) | 46.5 | 13.4 | | 23 | | |
| Senior high school teachers | 6.2 (5.3) | 54 (14) | 39.2 | 2.8 (0.9) | 57.3 | 136 (15) | 27.3 | 89.8 (19.5) | | 20.2 | | 29.9 (4.8) | 44.5 | 12.3 | | 21.5 | | |
| Managers of small enterprises | 6.6 (5.3) | 54 (14) | 43.6 | 2.8 (1.0) | 57.7 | 136 (15) | 27.8 | 90.5 (20.1) | | 21.2 | | 30.5 (4.8) | 48.4 | 16.2 | | 27.2 | | |
| Computer technicians and data operators | 5.9 (5.0) | 53 (14) | 38.0 | 2.7 (0.9) | 54.9 | 135 (15) | 24.1 | 90.4 (20.0) | | 18.4 | | 30.8 (5.1) | 51.3 | 15.3 | | 24.5 | | |
| Finance and sales associate professionals | 6.2 (5.2) | 54 (14) | 40.6 | 2.8 (0.9) | 55.9 | 136 (15) | 26.8 | 89.5 (20.3) | | 19 | | 30.1 (4.6) | 44.9 | 13.3 | | 22 | | |
| Computer scientists | 6.1 (5.1) | 52 (14) | 34.8 | 2.8 (0.9) | 58.8 | 134 (15) | 24.3 | 87.6 (19.4) | | 16.7 | | 30.4 (5.2) | 46.1 | 10.8 | | 21.6 | | |
| Elementary school teacher | 6.6 (5.3) | 54 (14) | 40.0 | 2.8 (0.9) | 59.7 | 135 (16) | 24.9 | 91.3 (20.7) | | 20.8 | | 29.5 (4.8) | 40.7 | 14.2 | | 19.9 | | |
| College and university teachers | 6.6 (5.3) | 51 (13) | 29.7 | 2.7 (0.9) | 57.3 | 136 (17) | 27.7 | 86.1 (18.6) | | 18.2 | | 29.2 (4.9) | 34.6 | 10.3 | | 20 | | |
| Directors and chief executives | 6.6 (5.2) | 53 (13) | 40.5 | 2.8 (0.9) | 60.0 | 136 (15) | 28.0 | 89.5 (19.9) | | 19.3 | | 30.3 (4.7) | 44.3 | 12.5 | | 22.6 | | |
| Business professionals | 6.4 (5.3) | 53 (14) | 37.6 | 2.7 (0.9) | 56.4 | 136 (15) | 27.8 | 88.1 (19.3) | | 18.2 | | 29.8 (4.7) | 42 | 11.8 | | 19.9 | | |
| Production and operations managers | 6.2 (5.0) | 53 (13) | 41.2 | 2.8 (0.9) | 56.3 | 136 (15) | 28.4 | 89.2 (19.1) | | 18.7 | | 30.2 (4.5) | 46.9 | 12.7 | | 20.4 | | |
| Architects and civil engineers | 6.3 (5.2) | 52 (13) | 35.3 | 2.8 (0.9) | 59.1 | 135 (15) | 26.7 | 87.4 (18.9) | | 19.1 | | 29.7 (4.7) | 42.7 | 11.4 | | 21.8 | | |
| Specialist managers | 6.1 (5.0) | 53 (13) | 38.4 | 2.8 (0.9) | 56.3 | 135 (15) | 26.1 | 88.4 (18.9) | | 19.6 | | 29.9 (4.6) | 42.5 | 10.0 | | 19.2 | | |
| The GFR was estimated with the use of the Modification of Diet in Renal Disease equation. Above target was defined according to current guidelines as >53 mmol/mol (7.0%) for HbA_1c_, >2.5 mmol/l for LDL-cholesterol, >140 mmhg for systolic blood pressure. Obesity was defined as BMI ≥30 kg/m^2.^ Albuminuria was defined as either microalbuminuria or macroalbuminuria. | | | | | | | | | | | | | | | | | |  |

Table S9. CVD risk factors in women with TYPE 2 DIABETES across the 30 most common occupational groups in Sweden 2002-2015.

|  | Diabetes duration (years) | HbA_1c_ (mmol/mol) | | | LDL-cholesterol (mmol/l) | | | Systolic blood pressure (mmhg) | | eGFR | Albumi-nuria | BMI (kg/m^2^) | | Smoking | Physical activity |
| --- | --- | --- | --- | --- | --- | --- | --- | --- | --- | --- | --- | --- | --- | --- | --- |
|  | **mean (sd)** | **mean (sd)** | **% above target** | **mean (sd)** | | **% above target** | **mean (sd)** | | **% above target** | **mean (sd)** | **%** | **Mean (sd)** | **% with obesity** | **% smokers** | **% active <30 min**  **/week** |
| Manufacturing labourers | 6.0 (5.1) | 54 (14) | 42.6 | 3.0 (0.9) | | 68.3 | 135 (17) | | 27.1 | 87.1 (22.3) | 16.8 | 31.9 (6.6) | 59.8 | 25.6 | 20 |
| Cleaners | 6.3 (5.5) | 54 (14) | 38.6 | 2.9 (1.0) | | 62.7 | 134 (16) | | 25.1 | 91.7 (24.2) | 16.9 | 30.9 (5.7) | 52.9 | 23.6 | 22.6 |
| Client information clerks | 5.9 (5.2) | 52 (13) | 34.1 | 3.0 (1.0) | | 64.2 | 134 (16) | | 24.7 | 86.5 (21.1) | 15.9 | 31.7 (6.1) | 57.5 | 20.9 | 22.1 |
| Cooks. waitresses and housekeepers | 6.4 (5.5) | 53 (14) | 38.7 | 3.0 (1.0) | | 63.4 | 135 (16) | | 27.4 | 91.3 (24.0) | 16.8 | 31.6 (5.8) | 57.0 | 21.0 | 21.8 |
| Total working population | 6.3 (5.3) | 53 (14) | 35.6 | 2.9 (1.0) | | 62.8 | 134 (16) | | 24.1 | 88.0 (21.9) | 14.1 | 31.3 (6.0) | 55.0 | 19.2 | 21.9 |
| Office clerks | 6.3 (5.3) | 52 (14) | 35.3 | 2.9 (1.0) | | 64.5 | 135 (16) | | 26.4 | 85.2 (21.1) | 14.6 | 31.7 (6.1) | 57.3 | 19.8 | 22.7 |
| Personal carers^b^ | 6.3 (5.3) | 53 (14) | 37.2 | 2.9 (1.0) | | 62.2 | 134 (16) | | 23.4 | 90.0 (22.7) | 14.7 | 31.6 (5.9) | 57.2 | 21.6 | 22.2 |
| Retail salespersons | 5.9 (5.1) | 52 (13) | 34.6 | 2.9 (1.0) | | 63.8 | 134 (15) | | 23.5 | 87.7 (21.4) | 13.3 | 30.8 (5.8) | 52.4 | 21.9 | 22 |
| Kitchen assistants | 6.4 (5.3) | 54 (14) | 40.0 | 2.9 (0.9) | | 61.5 | 134 (17) | | 25.9 | 91.2 (23.4) | 16.8 | 31.1 (5.8) | 54.6 | 20.4 | 23.1 |
| Psychologists and social workers | 6.1 (5.0) | 52 (13) | 33.7 | 3.0 (1.0) | | 66.7 | 133 (14) | | 21.9 | 85.0 (20.0) | 11.4 | 31.6 (6.1) | 56.9 | 14.6 | 19.8 |
| Office secretaries and data entry operators | 6.2 (5.3) | 52 (14) | 35.0 | 2.9 (1.0) | | 62.5 | 135 (16) | | 27.3 | 85.3 (19.8) | 13 | 31.1 (5.8) | 54.5 | 17.1 | 23.3 |
| Finance and sales associate professionals | 5.5 (4.8) | 51 (13) | 30.5 | 3.0 (0.9) | | 65.8 | 134 (16) | | 24.8 | 85.3 (19.4) | 11.4 | 30.9 (5.8) | 53.1 | 16.5 | 22.4 |
| Social work associate professionals^c^ | 6.1 (5.1) | 53 (14) | 36.9 | 3.0 (1.0) | | 65.4 | 132 (15) | | 20.5 | 88.5 (22.6) | 16.5 | 32.3 (6.3) | 62.1 | 19.6 | 24.2 |
| Accountants | 6.0 (5.3) | 52 (13) | 32.6 | 2.9 (1.0) | | 62.5 | 134 (15) | | 23.5 | 85.5 (20.1) | 12.6 | 31.1 (6.1) | 52.4 | 15.6 | 19.8 |
| Senior high school teachers | 6.3 (5.4) | 52 (13) | 34.7 | 2.9 (0.9) | | 62.3 | 133 (15) | | 21.1 | 85.8 (18.9) | 13 | 31.9 (6.2) | 59.2 | 11.4 | 21.6 |
| Numerical clerks | 6.0 (5.3) | 51 (13) | 32.8 | 2.9 (1.0) | | 62.4 | 135 (16) | | 25.6 | 84.2 (19.6) | 12.7 | 31.2 (5.9) | 54.2 | 18.0 | 22.8 |
| Nurses | 6.1 (5.2) | 51 (12) | 32.8 | 2.9 (1.0) | | 64.0 | 134 (16) | | 22.8 | 85.3 (19.9) | 12 | 31.2 (5.7) | 54.6 | 17.0 | 19.2 |
| Computer scientists | 5.9 (5.1) | 51 (14) | 31.0 | 3.0 (1.0) | | 68.3 | 133 (15) | | 20.3 | 87.3 (21.2) | 17.3 | 32.3 (6.1) | 60.8 | 13.8 | 19.3 |
| Engineers and technicians | 6.0 (4.9) | 52 (12) | 31.4 | 2.9 (0.9) | | 66.2 | 133 (16) | | 19.7 | 85.3 (19.5) | 14.7 | 30.6 (6.2) | 48.1 | 18.4 | 14.8 |
| Production and operations managers | 6.0 (5.1) | 52 (14) | 32.0 | 3.0 (1.0) | | 63.9 | 134 (14) | | 24.1 | 86.7 (19.2) | 13.5 | 32.0 (6.1) | 61.5 | 12.7 | 20.1 |
| Cashiers. tellers and related clerks | 6.1 (5.1) | 54 (14) | 38.0 | 2.9 (1.0) | | 65.0 | 136 (15) | | 27.3 | 86.0 (21.9) | 16.6 | 31.1 (5.7) | 52.3 | 21.1 | 26.3 |
| Specialist managers | 5.7 (4.8) | 52 (14) | 33.2 | 3.1 (1.0) | | 71.0 | 136 (17) | | 26.7 | 85.6 (19.8) | 12.3 | 31.3 (5.8) | 56.4 | 15.4 | 18.8 |
| Business professionals | 5.8 (4.9) | 51 (13) | 32.7 | 2.9 (0.9) | | 62.1 | 134 (15) | | 23.7 | 85.9 (19.0) | 11.5 | 31.3 (5.9) | 54.5 | 14.2 | 22.5 |
| Public service administrative professionals | 6.4 (5.3) | 52 (13) | 35.1 | 3.0 (1.0) | | 64.5 | 135 (15) | | 25.4 | 85.6 (18.9) | 12.3 | 31.3 (6.0) | 53.7 | 13.8 | 20.9 |
| Physiotherapists and dental hygienists | 6.1 (5.3) | 51 (13) | 28.6 | 3.0 (0.9) | | 66.9 | 133 (16) | | 20.4 | 86.6 (20.5) | 12.3 | 30.5 (6.1) | 50.3 | 11.5 | 17.3 |
| Preschool and recreation teachers | 6.1 (5.1) | 51 (12) | 30.1 | 3.0 (0.9) | | 65.1 | 134 (15) | | 21.9 | 88.8 (21.4) | 11.4 | 31.1 (5.8) | 54.1 | 11.3 | 17.4 |
| Midwifes and specialist nurses | 6.2 (5.2) | 51 (12) | 31.8 | 3.0 (1.0) | | 63.7 | 134 (16) | | 26.6 | 84.2 (18.8) | 11 | 30.8 (5.4) | 52.5 | 7.5 | 15.4 |
| Elementary school teacher | 5.9 (4.9) | 52 (14) | 34.4 | 3.0 (0.9) | | 66.6 | 133 (15) | | 20.0 | 87.3 (20.5) | 13.4 | 30.9 (6.2) | 52.0 | 10.1 | 19.7 |
| Managers of small enterprises | 6.0 (5.0) | 52 (13) | 33.8 | 3.0 (1.0) | | 67.6 | 135 (16) | | 24.6 | 86.2 (19.3) | 11.1 | 31.5 (6.0) | 53.8 | 13.5 | 19.9 |
| Health professionals | 6.6 (5.8) | 51 (13) | 28.5 | 2.9 (1.0) | | 63.3 | 130 (15) | | 16.9 | 85.5 (20.1) | 12 | 30.0 (5.5) | 44.7 | 5.1 | 18.8 |
| Writers and creative or performing artists | 5.8 (5.1) | 51 (13) | 31.3 | 3.0 (1.0) | | 62.2 | 132 (16) | | 19.9 | 84.8 (18.0) | 8.9 | 31.7 (6.4) | 60.8 | 12.7 | 18.1 |
| The GFR was estimated with the use of the Modification of Diet in Renal Disease equation. Above target was defined according to current guidelines as >53 mmol/mol (7.0%) for HbA_1c_, >2.5 mmol/l for LDL-cholesterol, >140 mmhg for systolic blood pressure. Obesity was defined as BMI ≥30 kg/m^2.^ Albuminuria was defined as either microalbuminuria or macroalbuminuria. | | | | | | | | | | | | | | | |

**Table S10. all cause and CVD mortality 2002-2005 across the 30 most common occupations in Swedish men.**

|  | All-cause mortality | | | | | | | CVD mortality | | | | |
| --- | --- | --- | --- | --- | --- | --- | --- | --- | --- | --- | --- | --- |
|  | **No. Cases** | **Person-years** | **Age stand Incidence per 100 000 (95% CI)** | **SIR**  **(95% CI)** | **Absolute**  **10-year risk at age 60** | **No. Cases** | **Age stand Incidence per 100 000**  **(95% CI)** | | **SIR**  **(95% CI)** | **Absolute**  **10-year risk at age 60** |  |  |
| Manufacturing labourers^1^ | 3 636 | 487 622 | 1 277 (1 231-1 322) | 1.78 (1.72-1.84) | 15.1 (14.3-16.0) | 1 246 | 450 (422-475) | | 2.11 (2.00-2.23) | 5.4 (4.9-6.0) |  |  |
| Machine operators^1^ | 1 473 | 384 351 | 847 (795-899) | 1.15 (1.10-1.21) | 10.5 (9.6-11.5) | 446 | 281 (251-313) | | 1.25 (1.14-1.37) | 3.6 (3.0-4.2) |  |  |
| Personal carers^1b^ | 3 233 | 859 873 | 825 (792-858) | 1.18 (1.14-1.22) | 10.5 (9.9-11.1) | 870 | 239 (221-257) | | 1.14 (1.07-1.22) | 3.0 (2.6-3.3) |  |  |
| Stores and transport clerks | 3 904 | 900 022 | 825 (797-855) | 1.13 (1.10-1.17) | 10.6 (10.0-11.2) | 1 210 | 267 (250-284) | | 1.23 (1.16-1.30) | 3.6 (3.2-4.0) |  |  |
| Motor-vehicle drivers^1^ | 7 609 | 1 603 064 | 821 (801-842) | 1.13 (1.11-1.16) | 10.8 (10.4-11.2) | 2 379 | 263 (251-274) | | 1.23 (1.18-1.28) | 3.4 (3.2-3.7) |  |  |
| Metal- and mineral-products machine operators^1^ | 3 268 | 764507 | 820 (792-852) | 1.13 (1.09-1.17) | 11.0 (10.4-11.7) | 981 | 263 (247-284) | | 1.19 (1.12-1.27) | 3.5 (3.1-3.9) |  |  |
| Metal moulders. welders and sheet-metal workers^1^ | 2 686 | 606 873 | 815 (781-848) | 1.14 (1.10-1.19) | 10.8 (10.0-11.6) | 738 | 237 (219-256) | | 1.10 (1.02-1.18) | 3.2 (2.8-3.7) |  |  |
| Agricultural and other mobile-plant operators^1^ | 2 606 | 569 518 | 781 (748-815) | 1.09 (1.05-1.13) | 10.5 (9.8-11.2) | 799 | 241 (223-259) | | 1.16 (1.08-1.24) | 3.0 (2.7-3.4) |  |  |
| Building finishers and related trades workers^1^ | 7 021 | 1 543 726 | 768 (747-788) | 1.06 (1.04-1.09) | 10.5 (10.1-10.9) | 2 110 | 239 (228-249) | | 1.10 (1.06-1.15) | 3.3 (3.0-3.5) |  |  |
| Assemblers^1^ | 2 385 | 664 860 | 766 (728-800) | 1.06 (1.02-1.11) | 9.8 (9.1-10.5) | 707 | 245 (225-266) | | 1.12 (1.04-1.21) | 3.1 (2.7-3.6) |  |  |
| Office clerks | 2 141 | 471 499 | 765 (730-799) | 1.06 (1.02-1.11) | 9.4 (8.8-10.1) | 669 | 243 (224-262) | | 1.15 (1.06-1.24) | 3.1 (2.7-3.5) |  |  |
| Machinery mechanics and fitters^1^ | 3 625 | 927 310 | 714 (690-738) | 0.98 (0.95-1.02) | 9.3 (8.8-9.9) | 1 044 | 219 (205-233) | | 0.99 (0.93-1.05) | 2.7 (2.4-3.0) |  |  |
| Construction workers^1^ | 5 974 | 1 500 680 | 712 (694-732) | 0.99 (0.97-1.02) | 10.0 (9.6-10.4) | 1 675 | 210 (200-221) | | 0.97 (0.92-1.02) | 3.1 (2.8-3.3) |  |  |
| Total working population | 101 458 | 23 157 642 | 700 (695-706) | 1.00 (0.99-1.01) | 8.7 (8.6-8.8) | 29 440 | 210 (207-213) | | 1.00 (0.99-1.01) | 2.6 (2.5-2.7) |  |  |
| Retail salespersons | 2 683 | 842 318 | 694 (665-727) | 0.94 (0.91-0.98) | 9.3 (8.7-9.9) | 794 | 216 (199-233) | | 1.00 (0.93-1.07) | 2.8 (2.5-3.2) |  |  |
| Electricians. tele and electronics repairers^1^ | 1 975 | 572 115 | 673 (639-705) | 0.91 (0.87-0.95) | 8.2 (7.6-8.9) | 570 | 207 (188-227) | | 0.92 (0.84-1.00) | 2.3 (2.0-2.7) |  |  |
| Accountants | 1 657 | 445 631 | 609 (580-639) | 0.84 (0.80-0.88) | 7.5 (6.9-8.1) | 491 | 184 (166-202) | | 0.86 (0.78-0.93) | 2.2 (1.8-2.5) |  |  |
| Engineers and technicians | 7 393 | 2 248 138 | 563 (550-577) | 0.77 (0.75-0.79) | 7.4 (7.1-7.7) | 2 101 | 163 (156-170) | | 0.76 (0.72-0.79) | 2.0 (1.9-2.2) |  |  |
| Managers of small enterprises | 4 257 | 1 330 571 | 548 (530-566) | 0.74 (0.72-0.76) | 7.1 (6.8-7.5) | 1 108 | 147 (138-157) | | 0.66 (0.63-0.70) | 1.9 (1.7-2.1) |  |  |
| Computer technicians and data operators | 1 059 | 524 087 | 548 (508-592) | 0.72 (0.68-0.77) | 6.9 (6.2-7.7) | 291 | 172 (147-202) | | 0.73 (0.65-0.82) | 2.1 (1.7-2.6) |  |  |
| Finance and sales associate professionals | 5 819 | 2 075 362 | 545 (530-562) | 0.73 (0.71-0.75) | 6.9 (6.6-7.2) | 1 481 | 142 (134-149) | | 0.65 (0.62-0.68) | 1.6 (1.5-1.8) |  |  |
| Public service administrative professionals | 1 879 | 504 238 | 540 (515-567) | 0.75 (0.72-0.79) | 6.9 (6.4-7.5) | 505 | 148 (134-162) | | 0.69 (0.63-0.76) | 2.0 (1.7-2.3) |  |  |
| Senior high school teachers | 1 743 | 502 636 | 520 (495-547) | 0.73 (0.70-0.76) | 6.5 (6.0-7.1) | 486 | 147 (134-162) | | 0.70 (0.64-0.76) | 1.8 (1.6-2.2) |  |  |
| Elementary school teacher | 1 153 | 368 270 | 520 (490-553) | 0.72 (0.68-0.77) | 6.4 (5.7-7.1) | 344 | 157 (140-176) | | 0.74 (0.67-0.83) | 1.9 (1.5-2.3) |  |  |
| Computer scientists | 1 912 | 1 066 566 | 481 (452-506) | 0.64 (0.62-0.67) | 5.8 (5.3-6.2) | 489 | 134 (120-149) | | 0.61 (0.55-0.66) | 1.6 (1.3-1.8) |  |  |
| Business professionals | 2 684 | 1 045 977 | 474 (455-495) | 0.64 (0.61-0.66) | 6.2 (5.8-6.6) | 742 | 137 (126-148) | | 0.61 (0.57-0.66) | 1.7 (1.5-1.9) |  |  |
| Production and operations managers | 2 827 | 1 065 092 | 463 (444-482) | 0.62 (0.60-0.64) | 5.9 (5.5-6.3) | 734 | 123 (114-133) | | 0.56 (0.52-0.60) | 1.4 (1.2-1.6) |  |  |
| Architects and civil engineers | 2 433 | 1 068 584 | 444 (424-464) | 0.60 (0.58-0.63) | 6.0 (5.6-6.4) | 633 | 121 (111-132) | | 0.55 (0.51-0.60) | 1.5 (1.3-1.7) |  |  |
| Specialist managers | 2 777 | 1 241 015 | 443 (425-462) | 0.58 (0.56-0.60) | 5.7 (5.3-6.1) | 725 | 120 (110-130) | | 0.53 (0.49-0.57) | 1.4 (1.2-1.6) |  |  |
| Directors and chief executives | 1 315 | 505 582 | 435 (410-462) | 0.59 (0.56-0.62) | 5.6 (5.1-6.1) | 312 | 103 (91-116) | | 0.48 (0.43-0.54) | 1.2 (1.0-1.5) |  |  |
| College and university teachers | 777 | 304 977 | 432 (401-466) | 0.61 (0.57-0.66) | 5.0 (4.4-5.8) | 214 | 123 (106-141) | | 0.59 (0.51-0.67) | 1.5 (1.1-1.9) |  |  |
| ^1^ Occupations classified as being in the lowest socio-economic status group. i.e. “skilled and unskilled manual workers” according to Statistics Sweden. ^b^Personal carers includes child-care workers, assistant nurses, hospital ward assistants, home-based personal care, attendants in psychiatric care and dental nurses. | | | | | | | | | | | |  |

**Table S11. ischemic heart disease (IHD) and stroke 2002-2005 across the 30 most common occupations in Swedish men.**

|  |  | IHD | | | | | | STROKE | | | |  |
| --- | --- | --- | --- | --- | --- | --- | --- | --- | --- | --- | --- | --- |
|  | | **No. Cases** | **Person-years** | **Age stand Incidence per 100 000 (95% CI)** | **SIR**  **(95% CI)** | **Absolute**  **10-year risk at age 60** | **No. Cases** | | **Age stand Incidence per 100 000**  **(95% CI)** | **SIR**  **(95% CI)** | **Absolute**  **10-year risk at age 60** |  |
| Manufacturing labourers^1^ | | 4 590 | 487 622 | 1 484 (1 420-1 549) | 1.39 (1.35-1.43) | 13.9 (13.0-15.0) | 1 546 | | 528 (496-560) | 1.45 (1.38-1.53) | 6.7 (6.0-7.3) |  |
| Motor-vehicle drivers^1^ | | 13 767 | 1 603 064 | 1 353 (1 319-1 387) | 1.27 (1.25-1.29) | 13.9 (13.4-14.4) | 4 373 | | 469 (454-485) | 1.25 (1.22-1.29) | 6.2 (5.8-6.5) |  |
| Agricultural and other mobile-plant operators^1^ | | 4 678 | 5 69 518 | 1 311 (1 254-1 366) | 1.22 (1.19-1.26) | 13.2 (12.3-14.0) | 1 476 | | 443 (417-472) | 1.18 (1.12-1.25) | 6.2 (5.6-6.8) |  |
| Personal carers^1b^ | | 5 494 | 859 873 | 1 301 (1 241-1 362) | 1.24 (1.20-1.27) | 13.2 (12.5-14.0) | 1 647 | | 430 (401-457) | 1.17 (1.12-1.23) | 5.5 (5.0-6.0) |  |
| Metal moulders. welders and sheet-metal workers^1^ | | 4 394 | 606 873 | 1 281 (1 224-1 337) | 1.17 (1.14-1.21) | 13.8 (13.0-14.7) | 1 278 | | 394 (368-421) | 1.05 (1.00-1.11) | 5.2 (4.7-5.7) |  |
| Machine operators^1^ | | 2 339 | 384 351 | 1 276 (1 193-1 362) | 1.15 (1.10-1.19) | 12.7 (11.6-13.8) | 721 | | 411 (373-452) | 1.09 (1.02-1.18) | 5.4 (4.7-6.2) |  |
| Building finishers and related trades workers^1^ | | 11 671 | 1 543 726 | 1 222 (1 186-1 256) | 1.10 (1.08-1.12) | 12.6 (12.1-13.1) | 3 703 | | 411 (396-428) | 1.08 (1.04-1.11) | 5.5 (5.2-5.9) |  |
| Stores and transport clerks^1^ | | 6 133 | 900 022 | 1 211 (1 163-1 261) | 1.11 (1.08-1.14) | 12.1 (11.4-12.8) | 1 918 | | 405 (383-427) | 1.07 (1.03-1.12) | 5.6 (5.2-6.1) |  |
| Machinery mechanics and fitters^1^ | | 6 357 | 927 310 | 1 190 (1 147-1 237) | 1.07 (1.05-1.10) | 12.3 (11.6-13.0) | 1 903 | | 378 (357-399) | 1.00 (0.95-1.04) | 5.1 (4.6-5.5) |  |
| Metal- and mineral-products machine operators^1^ | | 5 025 | 764 507 | 1 187 (1 141-1 233) | 1.09 (1.06-1.13) | 12.1 (11.4-12.8) | 1 603 | | 403 (379-427) | 1.08 (1.02-1.13) | 5.2 (4.8-5.7) |  |
| Assemblers^1^ | | 3 918 | 664 860 | 1 179 (1 124-1 237) | 1.09 (1.06-1.13) | 11.7 (10.9-12.4) | 1 277 | | 415 (386-445) | 1.11 (1.05-1.17) | 5.4 (4.9-6.0) |  |
| Office clerks | | 3 434 | 471 499 | 1 178 (1 117-1 237) | 1.07 (1.03-1.10) | 10.9 (10.1-11.7) | 1 099 | | 382 (358-410) | 1.04 (0.98-1.11) | 4.6 (4.1-5.1) |  |
| Retail salespersons | | 4 833 | 842 318 | 1 167 (1 114-1 221) | 1.07 (1.04-1.10) | 12.2 (11.5-13.0) | 1 536 | | 396 (371-423) | 1.05 (1.00-1.11) | 5.0 (4.6-5.5) |  |
| Construction workers^1^ | | 9 438 | 1 500 680 | 1 100 (1 070-1 131) | 0.98 (0.96-1.00) | 12.1 (11.6-12.6) | 3 107 | | 378 (362-394) | 1.00 (0.96-1.03) | 5.3 (4.9-5.6) |  |
| Total working population | | 160 891 | 23 157 642 | 1 094 (1 085-1 103) | 1.00 (1.00-1.01) | 11.2 (11.1-11.3) | 52 705 | | 373 (369-377) | 1.00 (0.99-1.01) | 4.7 (4.7-4.8) |  |
| Electricians. tele and electronics repairers^1^ | | 3 464 | 572 115 | 1 086 (1 031-1 141) | 0.98 (0.95-1.01) | 11.0 (10.3-11.8) | 1 077 | | 360 (333-387) | 0.95 (0.90-1.01) | 4.4 (3.9-4.9) |  |
| Managers of small enterprises | | 8 668 | 1 330 571 | 1 053 (1 019-1 092) | 0.93 (0.91-0.95) | 10.8 (10.3-11.3) | 2 707 | | 344 (327-360) | 0.90 (0.86-0.93) | 4.2 (3.9-4.5) |  |
| Engineers and technicians | | 13 401 | 2 248 138 | 984 (961-1 008) | 0.87 (0.86-0.89) | 10.1 (9.7-10.5) | 4 370 | | 330 (319-341) | 0.87 (0.85-0.90) | 4.2 (4.0-4.4) |  |
| Accountants | | 2 784 | 445 631 | 976 (924-1 027) | 0.87 (0.84-0.91) | 10.1 (9.3-11.0) | 983 | | 367 (338-397) | 0.95 (0.89-1.01) | 4.5 (4.0-5.0) |  |
| Computer technicians and data operators | | 2 019 | 524 087 | 974 (901-1 063) | 0.86 (0.83-0.90) | 10.2 (9.2-11.1) | 659 | | 301 (270-334) | 0.89 (0.82-0.96) | 4.0 (3.5-4.6) |  |
| Finance and sales associate professionals | | 10 897 | 2 075 362 | 974 (948-1 004) | 0.85 (0.84-0.87) | 9.8 (9.4-10.2) | 3 511 | | 321 (308-334) | 0.85 (0.82-0.88) | 4.0 (3.7-4.2) |  |
| Elementary school teacher | | 2 171 | 368 270 | 946 (889-1 010) | 0.85 (0.81-0.89) | 10.1 (9.2-11.1) | 711 | | 313 (287-341) | 0.86 (0.79-0.92) | 4.1 (3.5-4.7) |  |
| Senior high school teachers | | 3 171 | 502 636 | 928 (876-982) | 0.83 (0.80-0.86) | 10.1 (9.4-11.1) | 1 095 | | 330 (307-354) | 0.88 (0.83-0.93) | 4.4 (3.9-4.9) |  |
| Production and operations managers | | 5 942 | 1 065 092 | 911 (879-945) | 0.80 (0.78-0.82) | 9.4 (8.9-9.9) | 1 892 | | 305 (286-321) | 0.79 (0.76-0.83) | 3.6 (3.3-3.9) |  |
| Public service administrative professionals | | 3 168 | 504 238 | 894 (853-939) | 0.80 (0.77-0.82) | 9.2 (8.6-9.9) | 1 107 | | 316 (293-337) | 0.84 (0.80-0.90) | 4.0 (3.6-4.4) |  |
| Business professionals | | 5 032 | 1 045 977 | 859 (824-900) | 0.74 (0.72-0.76) | 8.8 (8.3-9.3) | 1 633 | | 283 (268-300) | 0.74 (0.71-0.78) | 3.3 (3.1-3.6) |  |
| Directors and chief executives | | 2 725 | 505 582 | 848 (796-903) | 0.75 (0.72-0.78) | 8.8 (8.1-9.5) | 862 | | 284 (261-305) | 0.74 (0.69-0.79) | 3.6 (3.2-4.0) |  |
| Specialist managers | | 5 623 | 1 241 015 | 827 (797-859) | 0.72 (0.70-0.74) | 8.6 (8.1-9.1) | 1 806 | | 276 (260-291) | 0.72 (0.69-0.76) | 3.5 (3.2-3.8) |  |
| Architects and civil engineers | | 4 388 | 1 068 584 | 802 (770-836) | 0.69 (0.67-0.71) | 8.8 (8.2-9.4) | 1 453 | | 265 (250-282) | 0.70 (0.66-0.73) | 3.2 (2.9-3.5) |  |
| Computer scientists | | 3 298 | 1 066 566 | 801 (754-846) | 0.70 (0.67-0.72) | 8.3 (7.7-8.9) | 1 141 | | 295 (272-320) | 0.76 (0.71-0.80) | 4.1 (3.6-4.5) |  |
| College and university teachers | | 1 196 | 304 977 | 687 (637-742) | 0.60 (0.57-0.64) | 7.7 (6.8-8.7) | 447 | | 264 (237-292) | 0.68 (0.62-0.75) | 3.4 (2.8-4.0) |  |
| ^1^ Occupations classified as being in the lowest socio-economic status group. i.e. “skilled and unskilled manual workers” according to Statistics Sweden. ^b^Personal carers includes child-care workers, assistant nurses, hospital ward assistants, home-based personal care, attendants in psychiatric care and dental nurses. | | | | | | | | | | | | |

**Table S12. all cause and CVD mortality 2002-2005 across the 30 most common occupations in Swedish women.**

|  | All cause-mortality | | | | | |  |  | | CVD-mortality | | |
| --- | --- | --- | --- | --- | --- | --- | --- | --- | --- | --- | --- | --- |
|  | No. Cases | Person-years | Age stand Incidence per 100 000 (95% CI) | SIR  (95% CI) | Absolute  10-year risk at age 60 | No. Cases | | | Age stand Incidence per 100 000 (95% CI) | | SIR  (95% CI) | Absolute  10-year risk at age 60 |
| Manufacturing labourers^1^ | 1 658 | 326 591 | 821 (779-864) | 1.76 (1.67-1.85) | 10.5 (9.7-11.5) | 393 | | | 201 (180-222) | | 2.63 (2.37-2.90) | 2.5 (2.0-3.0) |
| Cleaners^1^ | 3 938 | 1 130 828 | 566 (547-585) | 1.22 (1.19-1.26) | 7.6 (7.2-8.0) | 785 | | | 117 (108-126) | | 1.52 (1.42-1.63) | 1.5 (1.3-1.7) |
| Kitchen assistants^1^ | 2 777 | 828 027 | 522 (503-543) | 1.13 (1.09-1.18) | 7.3 (6.8-7.8) | 520 | | | 103 (93-112) | | 1.31 (1.20-1.43) | 1.6 (1.3-1.8) |
| Cooks, waitresses and housekeepers^1^ | 1 898 | 619 575 | 505 (481-529) | 1.09 (1.04-1.14) | 6.8 (6.3-7.4) | 346 | | | 98 (86-108) | | 1.24 (1.11-1.38) | 1.4 (1.1-1.7) |
| Cashiers. tellers and related clerks^1^ | 1 064 | 333 823 | 484 (457-516) | 1.06 (0.99-1.12) | 6.6 (5.8-7.4) | 164 | | | 80 (67-94) | | 1.02 (0.87-1.19) | 1.4 (1.1-1.8) |
| Personal carers^1b^ | 17 377 | 6 320 455 | 479 (471-487) | 1.04 (1.02-1.05) | 6.3 (6.2-6.5) | 2913 | | | 86 (83-90) | | 1.11 (1.07-1.15) | 1.1 (1.1-1.2) |
| Office clerks | 5 620 | 1 812 027 | 470 (458-484) | 1.01 (0.99-1.04) | 6.2 (5.9-6.5) | 935 | | | 82 (77-88) | | 1.05 (0.98-1.12) | 1.1 (1.0-1.2) |
| Client information clerks | 1 566 | 616 467 | 470 (447-495) | 1.00 (0.95-1.05) | 6.0 (5.5-6.5) | 237 | | | 77 (67-87) | | 0.97 (0.85-1.10) | 0.9 (0.7-1.2) |
| Total working population | 66 117 | 22 552 280 | 452 (448-456) | 1.00 (0.99-1.01) | 5.6 (5.5-5.7) | 10641 | | | 77 (75-78) | | 1.00 (0.98-1.02) | 1.0 (0.9-1.0) |
| Retail salespersons^1^ | 3 675 | 1 475 487 | 437 (423-453) | 0.94 (0.91-0.97) | 6.2 (5.8-6.5) | 609 | | | 76 (70-82) | | 0.97 (0.90-1.05) | 1.0 (0.9-1.2) |
| Social work associate professionals^c^ | 945 | 429 639 | 431 (400-464) | 0.92 (0.86-0.98) | 5.6 (4.9-6.3) | 143 | | | 72 (58-85) | | 0.91 (0.77-1.07) | 0.8 (0.6-1.0) |
| Numerical clerks | 3 345 | 1 217 159 | 429 (413-444) | 0.91 (0.88-0.94) | 5.7 (5.4-6.1) | 513 | | | 70 (63-76) | | 0.88 (0.80-0.96) | 0.9 (0.7-1.0) |
| Office secretaries and data entry operators | 2 607 | 904 515 | 425 (408-441) | 0.92 (0.88-0.95) | 5.7 (5.3-6.1) | 390 | | | 66 (59-72) | | 0.86 (0.77-0.95) | 0.8 (0.7-1.0) |
| Finance and sales associate professionals | 2 305 | 1 233 186 | 403 (384-421) | 0.83 (0.79-0.86) | 5.2 (4.9-5.6) | 301 | | | 63 (55-71) | | 0.72 (0.64-0.80) | 0.7 (0.6-0.9) |
| Accountants | 3 280 | 1 389 753 | 396 (381-411) | 0.83 (0.80-0.86) | 5.2 (4.9-5.5) | 445 | | | 59 (53-65) | | 0.72 (0.66-0.79) | 0.8 (0.7-0.9) |
| Computer scientists | 483 | 357 010 | 387 (342-437) | 0.80 (0.73-0.87) | 5.0 (4.2-5.8) | 52 | | | 61 (40-85) | | 0.62 (0.46-0.81) | 0.7 (0.4-1.1) |
| Engineers and technicians | 737 | 420 935 | 383 (352-413) | 0.78 (0.72-0.84) | 4.6 (4.0-5.2) | 105 | | | 62 (50-78) | | 0.74 (0.60-0.89) | 0.6 (0.4-0.9) |
| Psychologists and social workers | 1 039 | 452 791 | 379 (353-405) | 0.82 (0.77-0.87) | 4.7 (4.2-5.2) | 125 | | | 51 (41-61) | | 0.63 (0.52-0.75) | 0.7 (0.5-0.9) |
| Managers of small enterprises | 1 259 | 599 345 | 373 (350-395) | 0.78 (0.73-0.82) | 5.0 (4.6-5.5) | 174 | | | 56 (47-66) | | 0.69 (0.59-0.80) | 0.7 (0.5-0.9) |
| Public service administrative professionals | 1 516 | 684 266 | 371 (351-392) | 0.79 (0.75-0.83) | 5.1 (4.6-5.5) | 204 | | | 55 (47-63) | | 0.68 (0.59-0.78) | 0.7 (0.5-0.9) |
| Nurses | 1 927 | 973 746 | 361 (343-380) | 0.77 (0.74-0.81) | 4.7 (4.3-5.0) | 251 | | | 52 (45-59) | | 0.65 (0.57-0.74) | 0.7 (0.6-0.9) |
| Business professionals | 1 895 | 1 097 773 | 360 (343-379) | 0.74 (0.71-0.77) | 4.6 (4.3-5.0) | 238 | | | 51 (45-59) | | 0.62 (0.54-0.70) | 0.6 (0.5-0.8) |
| Writers and creative or performing artists | 581 | 359 744 | 349 (319-384) | 0.75 (0.69-0.82) | 4.4 (3.8-5.0) | 60 | | | 42 (31-55) | | 0.52 (0.39-0.66) | 0.4 (0.3-0.7) |
| Senior high school teachers | 1 268 | 579 873 | 340 (320-359) | 0.73 (0.69-0.77) | 4.2 (3.8-4.7) | 159 | | | 45 (38-52) | | 0.57 (0.49-0.67) | 0.6 (0.4-0.8) |
| Production and operations managers | 1 066 | 593 718 | 339 (316-364) | 0.69 (0.65-0.73) | 4.0 (3.6-4.4) | 115 | | | 42 (33-50) | | 0.49 (0.41-0.59) | 0.5 (0.3-0.7) |
| Preschool and recreation teachers | 1 765 | 1 133 050 | 332 (314-351) | 0.69 (0.66-0.72) | 4.1 (3.7-4.4) | 197 | | | 43 (36-50) | | 0.52 (0.45-0.60) | 0.6 (0.5-0.8) |
| Specialist managers | 713 | 492 005 | 329 (302-360) | 0.67 (0.62-0.72) | 4.2 (3.7-4.7) | 92 | | | 56 (43-70) | | 0.59 (0.48-0.73) | 0.6 (0.4-0.8) |
| Physiotherapists and dental hygienists | 860 | 466 369 | 323 (298-346) | 0.71 (0.66-0.75) | 4.2 (3.8-4.8) | 93 | | | 38 (30-46) | | 0.49 (0.40-0.60) | 0.5 (0.3-0.7) |
| Elementary school teacher | 2 100 | 1 099 999 | 321 (308-337) | 0.70 (0.67-0.73) | 3.8 (3.5-4.1) | 253 | | | 42 (37-48) | | 0.53 (0.47-0.60) | 0.5 (0.4-0.6) |
| Midwifes and specialist nurses | 1 296 | 662 911 | 312 (293-332) | 0.67 (0.64-0.71) | 3.8 (3.5-4.2) | 141 | | | 37 (30-44) | | 0.47 (0.39-0.55) | 0.5 (0.3-0.6) |
| Health professionals | 529 | 328 457 | 308 (278-339) | 0.65 (0.60-0.71) | 3.5 (3.0-4.1) | 51 | | | 35 (24-45) | | 0.41 (0.30-0.54) | 0.4 (0.2-0.6) |
| ^1^ Occupations classified as being in the lowest socio-economic status group. i.e. “skilled and unskilled manual workers” according to Statistics Sweden. ^b^Personal carers includes child-care workers, assistant nurses, hospital ward assistants, home-based personal care, attendants in psychiatric care and dental nurses. ^c^Social work associate professionals includes treatment assistants and youth recreations leaders. For some occupations, confidence intervals could not be estimated as the number of cases was too small. | | | | | | | | | | | | |

**Table S13. ischemic heart disease (IHD) and stroke 2002-2005 across the 30 most common occupations in Swedish women.**

|  | |  | | IHD | | | | | Stroke | | | | |  |
| --- | --- | --- | --- | --- | --- | --- | --- | --- | --- | --- | --- | --- | --- | --- |
|  | **No. Cases** | | **Person-years** | | **Age stand Incidence per 100 000 (95% CI)** | **SIR**  **(95% CI)** | **Absolute**  **10-year risk at age 60** | **No. Cases** | | **Age stand Incidence per 100 000 (95% CI)** | **SIR**  **(95% CI)** | **Absolute**  **10-year risk at age 60** |  |  |
| Manufacturing labourers^1^ | 1 208 | | 326 591 | | 539 (497-587) | 1.63 (1.54-1.73) | 5.2 (4.5-5.9) | 677 | | 326 (299-353) | 1.70 (1.58-1.83) | 3.8 (3.2-4.4) |  |  |
| Cleaners^1^ | 3 154 | | 1 130 828 | | 431 (408-453) | 1.26 (1.22-1.30) | 4.6 (4.2-5.0) | 1640 | | 234 (220-248) | 1.20 (1.14-1.26) | 3.1 (2.8-3.5) |  |  |
| Personal carers^1^ | 15 983 | | 6 320 455 | | 423 (412-434) | 1.22 (1.20-1.24) | 4.5 (4.3-4.6) | 7953 | | 220 (215-227) | 1.13 (1.10-1.15) | 2.9 (2.8-3.0) |  |  |
| Social work associate professionals^c^ | 906 | | 429 639 | | 407 (363-454) | 1.14 (1.06-1.21) | 4.1 (3.4-4.7) | 429 | | 194 (169-218) | 0.99 (0.90-1.09) | 2.4 (2.0-2.8) |  |  |
| Kitchen assistants^1^ | 2 154 | | 828 027 | | 394 (369-425) | 1.13 (1.09-1.18) | 4.6 (4.2-5.1) | 1209 | | 222 (206-237) | 1.16 (1.10-1.23) | 2.9 (2.6-3.2) |  |  |
| Cooks, waitresses and housekeepers^1^ | 1 459 | | 619 575 | | 381 (355-407) | 1.08 (1.02-1.13) | 4.2 (3.7-4.7) | 799 | | 214 (197-232) | 1.08 (1.01-1.16) | 2.8 (2.4-3.2) |  |  |
| Retail salespersons^1^ | 3 257 | | 1 475 487 | | 381 (361-402) | 1.08 (1.05-1.12) | 4.3 (3.9-4.7) | 1683 | | 198 (187-210) | 1.01 (0.96-1.06) | 2.7 (2.5-3.0) |  |  |
| Office clerks | 4 450 | | 1 812 027 | | 360 (346-376) | 1.03 (1.00-1.06) | 3.7 (3.4-3.9) | 2317 | | 192 (183-202) | 0.98 (0.94-1.02) | 2.5 (2.3-2.7) |  |  |
| Cashiers. tellers and related clerks^1^ | 791 | | 333 823 | | 348 (314-379) | 1.01 (0.94-1.08) | 3.7 (3.2-4.3) | 457 | | 206 (182-229) | 1.07 (0.97-1.17) | 2.7 (2.2-3.2) |  |  |
| Total working population | 51 534 | | 22 552 280 | | 348 (343-353) | 1.00 (0.99-1.01) | 3.6 (3.5-3.7) | 28068 | | 195 (192-198) | 1.00 (0.99-1.01) | 2.5 (2.4-2.5) |  |  |
| Client information clerks | 1 203 | | 616 467 | | 335 (306-363) | 1.00 (0.94-1.05) | 3.3 (2.8-3.7) | 700 | | 212 (190-232) | 1.06 (0.98-1.14) | 2.9 (2.5-3.3) |  |  |
| Managers of small enterprises | 1 066 | | 599 345 | | 318 (290-345) | 0.85 (0.80-0.90) | 3.1 (2.7-3.5) | 625 | | 184 (167-204) | 0.92 (0.85-0.99) | 2.5 (2.1-2.9) |  |  |
| Numerical clerks | 2 500 | | 1 217 159 | | 310 (294-329) | 0.87 (0.84-0.91) | 3.1 (2.9-3.4) | 1342 | | 171 (160-181) | 0.87 (0.82-0.91) | 2.2 (2.0-2.5) |  |  |
| Nurses | 1 660 | | 973 746 | | 305 (282-331) | 0.85 (0.81-0.89) | 3.1 (2.8-3.5) | 852 | | 167 (153-181) | 0.82 (0.76-0.87) | 2.3 (2.0-2.6) |  |  |
| Office secretaries and data entry operators | 1 907 | | 904 515 | | 294 (275-313) | 0.86 (0.82-0.90) | 2.9 (2.7-3.2) | 1059 | | 172 (161-185) | 0.88 (0.83-0.94) | 2.2 (2.0-2.5) |  |  |
| Psychologists and social workers | 781 | | 452 791 | | 281 (249-314) | 0.79 (0.73-0.85) | 2.9 (2.4-3.4) | 470 | | 178 (161-198) | 0.89 (0.81-0.97) | 2.1 (1.8-2.5) |  |  |
| Finance and sales associate professionals | 1 586 | | 1 233 186 | | 279 (259-300) | 0.74 (0.70-0.77) | 2.7 (2.4-3.0) | 1022 | | 188 (174-202) | 0.87 (0.82-0.93) | 2.4 (2.1-2.7) |  |  |
| Preschool and recreation teachers | 1 424 | | 1 133 050 | | 279 (256-302) | 0.71 (0.68-0.75) | 3.1 (2.8-3.5) | 805 | | 152 (139-167) | 0.76 (0.71-0.82) | 1.8 (1.6-2.1) |  |  |
| Production and operations managers | 821 | | 593 718 | | 269 (242-298) | 0.69 (0.64-0.74) | 2.5 (2.2-2.9) | 496 | | 160 (143-179) | 0.77 (0.70-0.84) | 1.8 (1.5-2.1) |  |  |
| Accountants | 2 243 | | 1 389 753 | | 266 (250-281) | 0.73 (0.70-0.76) | 2.6 (2.3-2.8) | 1354 | | 167 (156-177) | 0.82 (0.77-0.86) | 2.1 (1.9-2.3) |  |  |
| Public service administrative professionals | 1 082 | | 684 266 | | 263 (241-286) | 0.73 (0.68-0.77) | 2.7 (2.4-3.1) | 653 | | 173 (157-189) | 0.81 (0.75-0.87) | 2.2 (1.9-2.5) |  |  |
| Midwifes and specialist nurses | 1 111 | | 662 911 | | 262 (240-285) | 0.73 (0.69-0.78) | 2.7 (2.3-3.0) | 559 | | 140 (125-156) | 0.69 (0.64-0.75) | 2.1 (1.8-2.4) |  |  |
| Engineers and technicians | 490 | | 420 935 | | 250 (220-282) | 0.68 (0.62-0.74) | 2.6 (2.2-3.0) | 331 | | 178 (154-201) | 0.84 (0.75-0.93) | 2.6 (2.1-3.2) |  |  |
| Business professionals | 1 257 | | 1 097 773 | | 245 (224-265) | 0.64 (0.60-0.67) | 2.5 (2.2-2.8) | 807 | | 157 (144-171) | 0.75 (0.70-0.81) | 2.0 (1.7-2.2) |  |  |
| Elementary school teacher | 1 594 | | 1 099 999 | | 242 (224-260) | 0.68 (0.65-0.71) | 2.4 (2.2-2.7) | 991 | | 154 (144-165) | 0.78 (0.73-0.83) | 2.0 (1.8-2.3) |  |  |
| Computer scientists | 292 | | 357 010 | | 242 (197-292) | 0.64 (0.57-0.72) | 2.8 (2.1-3.5) | 182 | | 169 (134-208) | 0.73 (0.63-0.84) | 2.0 (1.5-2.6) |  |  |
| Senior high school teachers | 901 | | 579 873 | | 237 (216-259) | 0.67 (0.62-0.71) | 2.8 (2.4-3.2) | 530 | | 144 (131-159) | 0.72 (0.66-0.78) | 1.8 (1.5-2.1) |  |  |
| Specialist managers | 486 | | 492 005 | | 230 (197-263) | 0.59 (0.54-0.65) | 2.3 (1.9-2.8) | 306 | | 149 (127-173) | 0.69 (0.62-0.77) | 2.2 (1.7-2.6) |  |  |
| Writers and creative or performing artists | 346 | | 359 744 | | 222 (191-257) | 0.59 (0.53-0.66) | 2.2 (1.7-2.7) | 232 | | 137 (114-160) | 0.72 (0.63-0.82) | 1.8 (1.4-2.3) |  |  |
| Physiotherapists and dental hygienists | 585 | | 466 369 | | 221 (193-252) | 0.61 (0.57-0.67) | 2.5 (2.0-3.1) | 384 | | 152 (134-171) | 0.75 (0.68-0.83) | 2.0 (1.6-2.4) |  |  |
| Health professionals | 395 | | 328 457 | | 214 (180-252) | 0.62 (0.56-0.69) | 1.7 (1.3-2.3) | 215 | | 134 (113-156) | 0.63 (0.55-0.73) | 1.8 (1.4-2.3) |  |  |
| ^1^ Occupations classified as being in the lowest socio-economic status group. i.e. “skilled and unskilled manual workers” according to Statistics Sweden. ^b^Personal carers includes child-care workers, assistant nurses, hospital ward assistants, home-based personal care, attendants in psychiatric care and dental nurses. ^c^Social work associate professionals includes treatment assistants and youth recreations leaders. For some occupations, confidence intervals could not be estimated as the number of cases was too small. | | | | | | | | | | | | | | |
